# High glutamine increases stroke risk by inducing the endothelial‐to‐mesenchymal transition in moyamoya disease

**DOI:** 10.1002/mco2.525

**Published:** 2024-04-15

**Authors:** Qiheng He, Junsheng Li, Chuming Tao, Chaofan Zeng, Chenglong Liu, Zhiyao Zheng, Siqi Mou, Wei Liu, Bojian Zhang, Xiaofan Yu, Yuanren Zhai, Jia Wang, Qian Zhang, Yan Zhang, Dong Zhang, Jizong Zhao, Peicong Ge

**Affiliations:** ^1^ Department of Neurosurgery Beijing Tiantan Hospital, Capital Medical University Beijing China; ^2^ Research Unit of Accurate Diagnosis, Treatment, and Translational Medicine of Brain Tumors Chinese Academy of Medical Sciences and Peking Union Medical College Beijing China; ^3^ Department of Neurosurgery Peking Union Medical College Hospital, Chinese Academy of Medical Sciences and Peking Union Medical College Beijing China; ^4^ 3D Printing Center in Clinical Neuroscience China National Clinical Research Center for Neurological Diseases Beijing China; ^5^ Department of Neurosurgery Beijing Hospital Beijing China

**Keywords:** endothelial‐to‐mesenchymal transition, glutamine, Integrin Subunit Beta 4, moyamoya, Smad, stroke

## Abstract

At present, there is limited research on the mechanisms underlying moyamoya disease (MMD). Herein, we aimed to determine the role of glutamine in MMD pathogenesis, and 360 adult patients were prospectively enrolled. Human brain microvascular endothelial cells (HBMECs) were subjected to Integrin Subunit Beta 4 (ITGB4) overexpression or knockdown and atorvastatin. We assessed factors associated with various signaling pathways in the context of the endothelial‐to‐mesenchymal transition (EndMT), and the expression level of related proteins was validated in the superficial temporal arteries of patients. We found glutamine levels were positively associated with a greater risk of stroke (OR = 1.599, *p* = 0.022). After treatment with glutamine, HBMECs exhibited enhanced proliferation, migration, and EndMT, all reversed by ITGB4 knockdown. In ITGB4‐transfected HBMECs, the MAPK–ERK–TGF–β/BMP pathway was activated, with Smad4 knockdown reversing the EndMT. Furthermore, atorvastatin suppressed the EndMT by inhibiting Smad1/5 phosphorylation and promoting Smad4 ubiquitination in ITGB4‐transfected HBMECs. We also found the protein level of ITGB4 was upregulated in the superficial temporal arteries of patients with MMD. In conclusion, our study suggests that glutamine may be an independent risk factor for hemorrhage or infarction in patients with MMD and targeting ITGB4 could potentially be therapeutic approaches for MMD.

## Graphical abstract

Glutamine levels were positively associated with a greater risk of stroke (OR = 1.599, *p* = 0.022). After treatment with glutamine, HBMECs exhibited enhanced proliferation, migration, and EndMT, all reversed by ITGB4 knockdown. In ITGB4‐transfected HBMECs, the MAPK–ERK–TGF–β/BMP pathway was activated, with Smad4 knockdown reversing the EndMT. Furthermore, atorvastatin suppressed the EndMT by inhibiting Smad1/5 phosphorylation and promoting Smad4 ubiquitination in ITGB4‐transfected HBMECs. Finally, ITGB4 upregulation was confirmed in the superficial temporal arteries of patients with MMD.

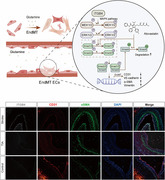

## 1 INTRODUCTION

Moyamoya disease (MMD) is a rare cerebrovascular condition characterized by progressive occlusion in the terminal portions of bilateral internal carotid arteries, which leads to the compensatory development of collateral vessels at the base of the brain.^1^, ^2^, ^3^, ^4^ In adult patients, MMD usually occurs at 30−40 years of age, with an annual incidence of 0.5−1.5 per 100,000 individuals in East Asian countries.^5^ Advances in diagnostic technologies have contributed to an annually increasing diagnostic rate of MMD. Although a rare cerebrovascular disease, MMD has become a common cause of stroke in children and young adults in China, associated with a high disability and mortality rate,^6^ causing a huge economic burden to families and society. Without treatment, most patients with MMD experience multiple strokes due to the progressive narrowing of arteries. These include ischemic stroke and hemorrhagic stroke, which can be fatal due to subsequent intracerebral hemorrhage or occlusion.^3^ Due to the unclear etiology of MMD, no specific therapeutic drugs are currently available. At present, the main treatment plan for patients with MMD is cerebral hemisphere revascularization, with the aim of restoring blood supply to the cerebral hemisphere, improving ischemic symptoms, and reducing the risk of recurrent stroke.^7^, ^8^, ^9^ However, some patients experience limited benefits after revascularization.^10^, ^11^ Thus, exploring the pathogenesis of and potential therapeutic targets for MMD is of great significance for preventing or reversing disease progression and improving long‐term prognosis.

Glutamine is a critical amino acid in the body, constituting up to 40% of the free amino acid pool in certain tissues.^12^, ^13^ Its biological significance has gradually been elucidated. Glutaminolysis involves a series of biochemical reactions that participate in several biological processes, including energy generation, nucleotide biosynthesis, and stress responses.^14^, ^15^, ^16^, ^17^, ^18^ A recent review on the *Signal Transduction and Targeted Therapy* ^19^ and other studies, including the PREDIMED trial, reported inadequate glutamine levels in ischemic stroke, ischemia–reperfusion injury, diabetes, and myocardial infarction,^20^, ^21^, ^22^, ^23^ suggesting a role for glutamine in cardiovascular or cerebrovascular disease. However, the possible involvement of glutamine in MMD progression has not yet been addressed.

The endothelial‐to‐mesenchymal transition (EndMT) is a process through which endothelial cells acquire mesenchymal phenotypes, while losing their endothelial characteristics.^24^ During the EndMT, certain endothelial markers may be lost or retained in parallel to the acquisition of mesenchymal markers and morphology.^25^, ^26^, ^27^, ^28^ Li et al.^29^, ^30^, ^31^, ^32^ recently reported that KRAS mutations regulate the EndMT in cerebral arteriovenous malformations, and KLF4 was described as a key determinant of the EndMT in cerebral cavernous malformations. Although the EndMT contributes to a variety of cerebrovascular disorders, its role in MMD remains unclear. Whether EndMT signaling can be targeted in patients with MMD requires further interrogation.

In this study, we used liquid chromatography–tandem mass spectrometry (LC–MS/MS) to detect serum glutamine levels in patients with MMD. Multivariate regression was then employed to analyze the relationship between glutamine levels and the manifestation of MMD upon admission. Further, we subjected human brain microvascular endothelial cells (HBMECs) to glutamine treatment or Integrin Subunit Beta 4 (ITGB4) overexpression in order to explore the mechanism of EndMT in MMD patients. We also assessed the efficacy of atorvastatin in reversing the EndMT in MMD. A deeper understanding of MMD pathophysiology is expected to contribute to the development of effective therapeutics against the disease.

## 2 RESULTS

### 2.1 High serum glutamine was independently associated with stroke risk in MMD

To determine whether serum glutamine levels were associated with stroke in MMD, we recruited 360 adult patients. Among them, 246 (68.33%) presented with stroke, including 145 (40.28%) with the infarction subtype and 101 (28.06%) with the hemorrhagic subtype (Table S1). The baseline characteristics and laboratory examinations of adult patients with MMD were subjected to univariate analysis, and were summarized in Table 1. Notably, the glutamine level in complete stroke group (3.51 [3.16–3.71]) was significantly higher than in the TIA group (3.64 [3.29–4.02], *p* = 0.008).

**TABLE 1:** Baseline characteristics of MMD patients.

Variables	All patients (*N* = 360)	TIA (*N* = 114)	Stroke (*N* = 246)	*p* Value
Age, y, median (IQR)	43.00 (34.00–50.00)	41.50 (34.00–48.00)	43.00 (33.50–50.50)	0.159
Sex, *n* (%)				0.909
Male	150 (41.67)	47 (31.33)	103 (68.70)	
Female	210 (58.33)	67 (31.90)	143 (68.10)	
History of risk factors, *n* (%)				
Hypertension	131 (36.39)	35 (26.72)	96 (73.28)	0.157
Diabetes mellitus	59 (16.39)	16 (27.12)	43 (72.88)	0.448
Hyperlipidemia	54 (15.00)	17 (31.48)	37 (68.52)	0.975
Cigarette smoking	71 (19.72)	20 (28.17)	51 (71.83)	0.569
Alcohol drinking	42 (11.67)	14 (33.33)	28 (66.67)	0.860
Clinical features, median (IQR)				
Heart rate (bpm)	78.00 (75.00–80.00)	78.00 (76.00–80.00)	78.00 (75.00–80.00)	0.534
SBP (mmHg)	132.00 (124.00–140.00)	133.00 (124.00–140.00)	132.00 (124.00–140.00)	0.516
DBP (mmHg)	81.00 (76.00–89.00)	80.00 (76.00–89.00)	81.00 (76.00–89.00)	0.747
BMI (kg/m^2^)	25.00 (22.49–27.78)	25.33 (22.77–28.09)	24.97 (22.49–27.76)	0.409
Laboratory results				
RBC (10^12^/L), mean ± SD	4.65 ± 0.51	4.69 ± 0.51	4.63 ± 0.51	0.265
HGB (g/L), mean ± SD	140.44 ± 18.32	143.04 ± 16.49	139.24 ± 19.03	0.067
HCT (L/L), median (IQR)	0.41 (0.38–0.45)	0.41 (0.39–0.45)	0.41 (0.38–0.45)	0.180
PLT count (10^9^/L), median (IQR)	248.00 (209.50–284.75)	249.50 (210.25–282.25)	247.00 (208.75–290.50)	0.883
Creatinine (µmol/L), median (IQR)	54.75 (46.33–66.98)	53.95 (46.63–66.68)	55.55 (46.20–67.33)	0.775
Uric acid (µmol/L), median (IQR)	4.50 (3.80–5.60)	4.50 (3.80–5.73)	4.55 (3.70–5.60)	0.711
TG (mmol/L), median (IQR)	1.20 (0.82–1.64)	1.24 (0.84–1.75)	1.18 (0.82–1.59)	0.346
TC (mmol/L), median (IQR)	4.23 (3.55–4.83)	4.23 (3.49–4.89)	4.23 (3.58–4.81)	0.967
HDL‐C (mmol/L), median (IQR)	1.30 (1.12–1.49)	1.31 (1.09–1.51)	1.30 (1.14–1.46)	0.928
LDL‐C (mmol/L), median (IQR)	2.40 (1.84–2.98)	2.36 (1.79–2.99)	2.40 (1.89–2.98)	0.579
ApoA_1_ (g/L), median (IQR)	1.29 (1.15–1.45)	1.32 (1.15–1.46)	1.29 (1.15–1.44)	0.407
ApoB (g/L), median (IQR)	0.82 (0.70–0.97)	0.84 (0.69–0.96)	0.82 (0.70–0.97)	0.791
Hcy (µmol/L), median (IQR)	12.00 (9.30–15.08)	11.11 (8.59–15.45)	12.10 (9.60–15.05)	0.221
HHcy, *n* (%)	91 (25.28)	29 (31.87)	62 (68.13)	0.773
Glutamine (10^3^ µmol/L), median (IQR)	3.58 (3.24–3.94)	3.51 (3.16–3.71)	3.64 (3.29–4.02)	0.008^*^

Abbreviations: ApoA_1_, apolipoprotein A_1_; ApoB, apolipoprotein B; BMI, body mass index; DBP, diastolic blood pressure; HDL‐C, high‐density lipoprotein cholesterol; Hcy, homocysteine; HHcy, hyperhomocysteinemia; LDL‐C, low‐density lipoprotein cholesterol; MMD, moyamoya disease; mRS, modified Rankin Scale; SBP, systolic blood pressure; SD, standard deviation; TIA, transient ischemic attack; TG, triglyceride; TC, total cholesterol; UA, uric acid.

^*^ *p* < 0.05, significant difference.

The characteristics of MMD patients subjected to glutamine levels are summarized in Table S2. When all the patients were stratified into tertiles based on their serum glutamine levels, there tended to be more male patients with the increase in glutamine levels (*p* < 0.001) and more patients with cigarette smoking (*p* = 0.006). No significant trend was found in age (*p* = 0.235), hypertension (*p* = 0.592), diabetes mellitus (*p* = 0.223), hyperlipidemia (*p* = 0.470), and alcohol drinking (*p* = 0.547). In laboratory examinations, patients with higher glutamine levels tended to have higher hemoglobin (HGB) (*p* = 0.006), hematocrit (HCT) (*p* = 0.012), creatinine (*p* < 0.001), homocysteine (Hcy) (*p* = 0.019), and hyperhomocysteinemia (HHcy) (*p* = 0.012).

To eliminate the influence of confounding factors on the association between glutamine and MMD, age, sex, heart rate, systolic blood pressure (SBP), diastolic blood pressure (DBP), body mass index (BMI), red blood cell (RBC) count, hemoglobin (HGB), hematocrit (HCT) value, platelet (PLT) count, triglyceride (TG), total cholesterol (TC), high‐density lipoprotein cholesterol (HDL‐C), low‐density lipoprotein cholesterol (LDL‐C), apolipoprotein A1 (ApoA_1_), apolipoprotein B (ApoB), and Hcy were adjusted for in the multivariate analysis. After adjusting for all variables, we still found a significantly higher risk of stroke in patients with each increment in glutamine levels (OR = 1.599, 95% CI = 1.017–2.387, *p* = 0.022). When patients were divided in tertiles based on glutamine levels, those with the highest glutamine level (T3, ≥3.80 mmol/L) had a higher risk of stroke than those with lower glutamine levels (T1‐2, <3.80 mmol/L) (OR = 2.752, 95% CI = 1.556–4.868, *p* = 0.001) (Table 2). Consistently, a significantly higher risk of infarction subtype of stroke (OR = 3.596, 95% CI = 1.894–6.827, *p* < 0.001) and a higher risk of hemorrhagic subtype of stroke (OR = 2.426, 95% CI = 1.153–5.104, *p* = 0.019) were found in patients in glutamine tertile 3 compared with those in tertile 1 to 2. These results indicate that high serum glutamine levels are independently associated with stroke risk.

**TABLE 2:** Association of glutamine levels and the risk of complete stroke.

		Crude model		Model 1^a^		Model 2^b^	
Glutamine (µmol/L)	Number of events, *n* (%)	OR (95%CI)	*p* Value	OR (95%CI)	*p* Value	OR (95%CI)	*p* Value
Stroke	246 (68.33)						
Continuous		1.564 (1.084–2.259)	0.017	1.617 (1.093–2.392)	0.016	1.599 (1.071–2.387)	0.022^*^
Tertiles							
T1 (<3.38)	77 (31.30)	ref.		ref.		ref.	
T2 (3.38–3.80)	72 (29.27)	0.838 (0.497–1.412)	0.506	0.852 (0.502–1.445)	0.553	0.848 (0.484–1.486)	0.563
T3 (>=3.80)	97 (39.43)	2.355 (1.308–4.241)	0.004	2.490 (1.339–4.631)	0.004	2.517 (1.319–4.803)	0.005^*^
T1‐2 (< 3.80)	149 (60.57)	ref.		ref.		ref.	
T3 (>=3.80)	97 (39.43)	2.576 (1.525–4.350)	<0.001	2.717 (1.569–4.704)	<0.001	2.752 (1.556–4.868)	0.001^*^
Infarction‐subtype	145 (55.98)						
Continuous		1.788 (1.170–2.733)	0.007	1.750 (1.113–2.752)	0.015	1.926 (1.189–3.120)	0.008^*^
Tertiles							
T1 (<3.38)	46 (31.72)	ref.		ref.		ref.	
T2 (3.38–3.80)	36 (24.83)	0.701 (0.385–1.277)	0.246	0.697 (0.379–1.281)	0.246	0.617 (0.312–1.220)	0.165
T3 (>=3.80)	63 (43.45)	2.560 (1.359–4.823)	0.004	2.486 (1.268–4.873)	0.008	2.820 (1.366–5.825)	0.005^*^
T1‐2 (<3.8)	82 (56.55)	ref.		ref.		ref.	
T3 (>=3.80)	63 (43.45)	3.040 (1.731–5.338)	<0.001	3.007 (1.663–5.437)	<0.001	3.596 (1.894–6.827)	<0.001^*^
Hemorrhagic‐subtype	101 (46.98)						
Continuous		1.386 (0.876–2.192)	0.163	1.567 (0.952–2.578)	0.077	1.652 (0.955–2.857)	0.073
Tertiles							
T1 (<3.38)	31 (30.69)	ref.		ref.		ref.	
T2 (3.38–3.80)	36 (35.64)	1.040 (0.553–1.958)	0.902	1.108 (0.579–2.122)	0.757	1.329 (0.636–2.777)	0.450
T3 (>=3.80)	34 (33.66)	2.050 (1.016–4.139)	0.045	2.500 (1.164–5.371)	0.019	2.856 (1.209–6.748)	0.017^*^
T1‐2 (<3.8)	67 (66.34)	ref.		ref.		ref.	
T3 (>=3.80)	34 (33.66)	2.008 (1.084–3.718)	0.027	2.353 (1.215–4.556)	0.011	2.426 (1.153–5.104)	0.019^*^

Abbreviations: CI, confidence interval; OR, odds ratio; TIA, transient ischemic attack.

^a^ Model 1, adjusted for age and sex.

^b^ Model 2, adjusted for age, sex, heart rate, SBP, DBP, BMI, RBC, HGB, TG, TC, HDL‐C, LDL‐C, ApoA_1_, ApoB, and Hcy.

^*^ *p* < 0.05, significant difference.

### 2.2 Glutamine promotes the proliferation, migration, and EndMT of HBMECs through ITGB4

**FIGURE 1:**
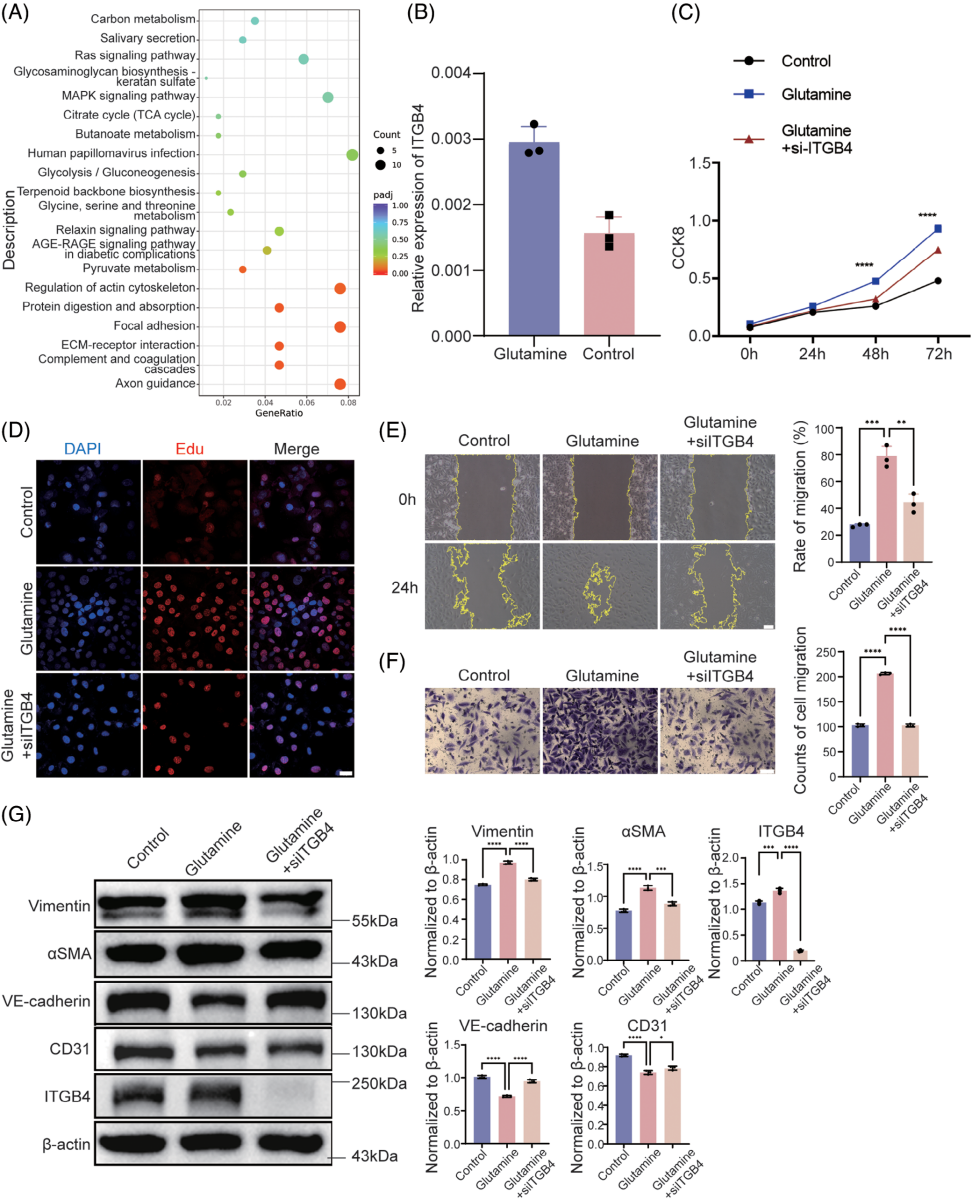
Glutamine promotes proliferation, migration, and EndMT through ITGB4 in HBMECs. (A) Kyoto Encyclopedia of Genes and Genomes enrichment analysis of HBMECs treated with glutamine. (B) RT‐qPCR analysis of ITGB4 expression in HBMECs treated with glutamine. (C) Proliferation ability of HBMECs subjected to glutamine treatment and ITGB4 knockdown assessed by CCK8 assay. (D) Representative images of EdU assays in HBMECs subjected to glutamine treatment and ITGB4 knockdown. Each image represents three replicates. Scale bar, 10 µm. (E) Representative images (left) and histogram (right) of HBMEC migration, as determined via wound healing assays. Each image represents three replicates. Scale bar in white, 100 µm. (F) Representative images (left) and histogram (right) of the migration ability of HBMECs, as determined via transwell assays. Each image represents three replicates. Scale bar in black, 25 µm. (G) Western blot analysis (left) and the histograms (right) of Vimentin, αSMA, VE‐cadherin, CD31, and ITGB4 in HBMECs subjected to different treatments. **p < 0.01, ***p < 0.001, ****p < 0.0001. EndMT, endothelial‐to‐mesenchymal transition; HBMECs, human brain microvascular endothelial cells.

Previous studies have suggested that endothelial cell dysfunction is an important pathological factor in patients with stroke. Therefore, we treated HBMECs with glutamine to explore its effect on endothelial cells. RNA‐sequencing (RNA‐seq) was performed to determine the downstream targets of glutamine. A total of 511 genes were significantly differentially regulated after glutamine treatment, of which 265 were upregulated and 246 were downregulated (Table S3). Furthermore, Kyoto Encyclopedia of Genes and Genomes pathway enrichment analysis was performed, yielding several enriched pathways (Figure 1A). Among these, the MAPK signaling pathway showed the highest gene ratio, while no pathway was significantly enriched. MAPK signaling has been reported as central to cell adhesion and migration, being potentially related to the progression of MMD. Among the significantly regulated genes implicated in the MAPK signaling pathway, we focused on ITGB4 (fold change = 1.52, *p* < 0.001), which was also significantly enriched in extracellular matrix (ECM)–receptor interaction (*P*adj = 0.044) and focal adhesion (*P*adj = 0.044; Figure 1A). We performed real time‐quantitative polymerase chain reaction (RT‐qPCR) to validate RNA‐seq ITGB4 expression results, confirming that ITGB4 levels were significantly upregulated in glutamine‐treated HBMECs (fold change = 1.90, *p* = 0.003; Figure 1B). To evaluate the proliferative ability of HBMECs, we performed CCK8 and EdU assays. After treatment with glutamine, HBMECs showed significantly enhanced proliferation based on both CCK8 and EdU assays (*p* < 0.05; Figures 1C, D, and S1). Moreover, small interfering RNA (siRNA)‐mediated ITGB4 knockdown significantly reversed glutamine‐induced proliferation (Figures 1C, D, and S2). Wound healing and transwell migration assays were performed to evaluate cellular behavior, with the results indicating enhanced migration of glutamine‐treated HBMECs compared with control HBMECs, which was also reversed by ITGB4 knockdown (Figures 1E and F). To further confirm the EndMT in HBMECs, we examined endothelial and mesenchymal markers at the protein level using western blotting. The expression of CD31 and VE‐cadherin was significantly downregulated, while Vimentin and αSMA were significantly upregulated after glutamine treatment, with ITGB4 knockdown reversing these changes (Figure 1G). The above‐described results suggested that glutamine promotes the proliferation, migration, and EndMT of HBMECs through ITGB4.

### 2.3 The activation of MAPK–ERK–TGF‐β/BMP, rather than Wnt or Notch signaling, contributes to ITGB4‐induced EndMT

**FIGURE 2:**
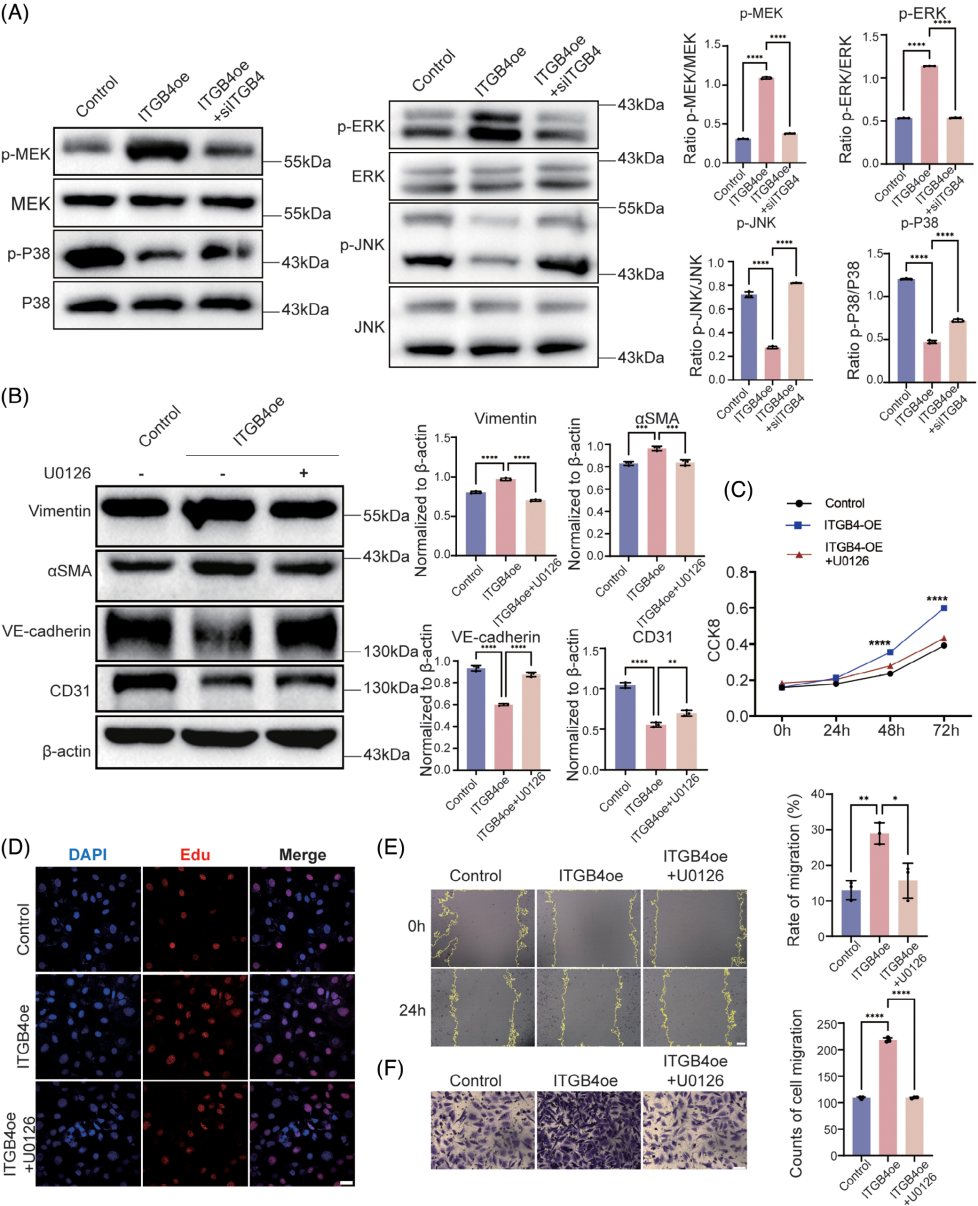
The activation of MAPK–ERK contributes to the ITGB4‐induced EndMT. Western blot assays (left) and histograms (right) show (A) significantly increased phosphorylation of MEK and ERK as well as decreased phosphorylation of JNK and P38. (B) Significant upregulation of mesenchymal markers Vimentin and αSMA with downregulation of endothelial markers VE‐cadherin and CD31. (C) Proliferation ability of ITGB4‐transfected HBMECs with or without MEK inhibitor U0126 treatment, assessed via CCK8 assay. (D) Representative images of proliferation ability of HBMECs subjected to different treatments assessed via EdU assays. Each image represents three replicates. Scale bar, 10 µm. (E) Representative images (left) and histogram (right) of HBMEC migration, as determined via wound healing assay. Each image represents three replicates. Scale bar, 100 µm. (F) Representative images (left) and histogram (right) of HBMEC migration, as determined via transwell assays. Each image represents three replicates. Scale bar in black, 25 µm. *p < 0.05, **p < 0.01, ***p < 0.001, ****p < 0.0001. EndMT, endothelial–mesenchymal transition; HBMECs, human brain microvascular endothelial cells.

ITGB4 was reported to play a critical role in the regulation of keratinocyte polarity and motility.^33^, ^34^ To further clarify the downstream signaling through which ITGB4 induces the EndMT, we examined the total levels and phosphorylation of MEK1/2, ERK1/2, JNK, and p38 MAPK in HBMECs transfected with ITGB4 adenovirus^35^ (Figure S3). We observed significantly increased MEK1/2 and ERK1/2 phosphorylation in ITGB4‐overexpressing HBMECs, without differences in JNK or p38 phosphorylation. Meanwhile, ITGB4 knockdown reversed the above‐described increase in MEK1/2 and ERK1/2 phosphorylation (Figure 2A). We treated ITGB4‐overexpressing HBMECs with specific MEK1/2 inhibitor U0126, observing a reversal of endothelial marker (CD31 and VE‐cadherin) downregulation and mesenchymal marker (Vimentin and αSMA) upregulation (Figure 2B). CCK8 and EdU assays showed a significantly enhanced proliferative ability under ITGB4 overexpression, while migration assays confirmed enhanced migration, both of which were reversed by U0126 treatment (Figures 2C–F and S4). These results suggest that ITGB4 induces the EndMT through MAPK–MEK–ERK signaling in HBMECs.

**FIGURE 3:**
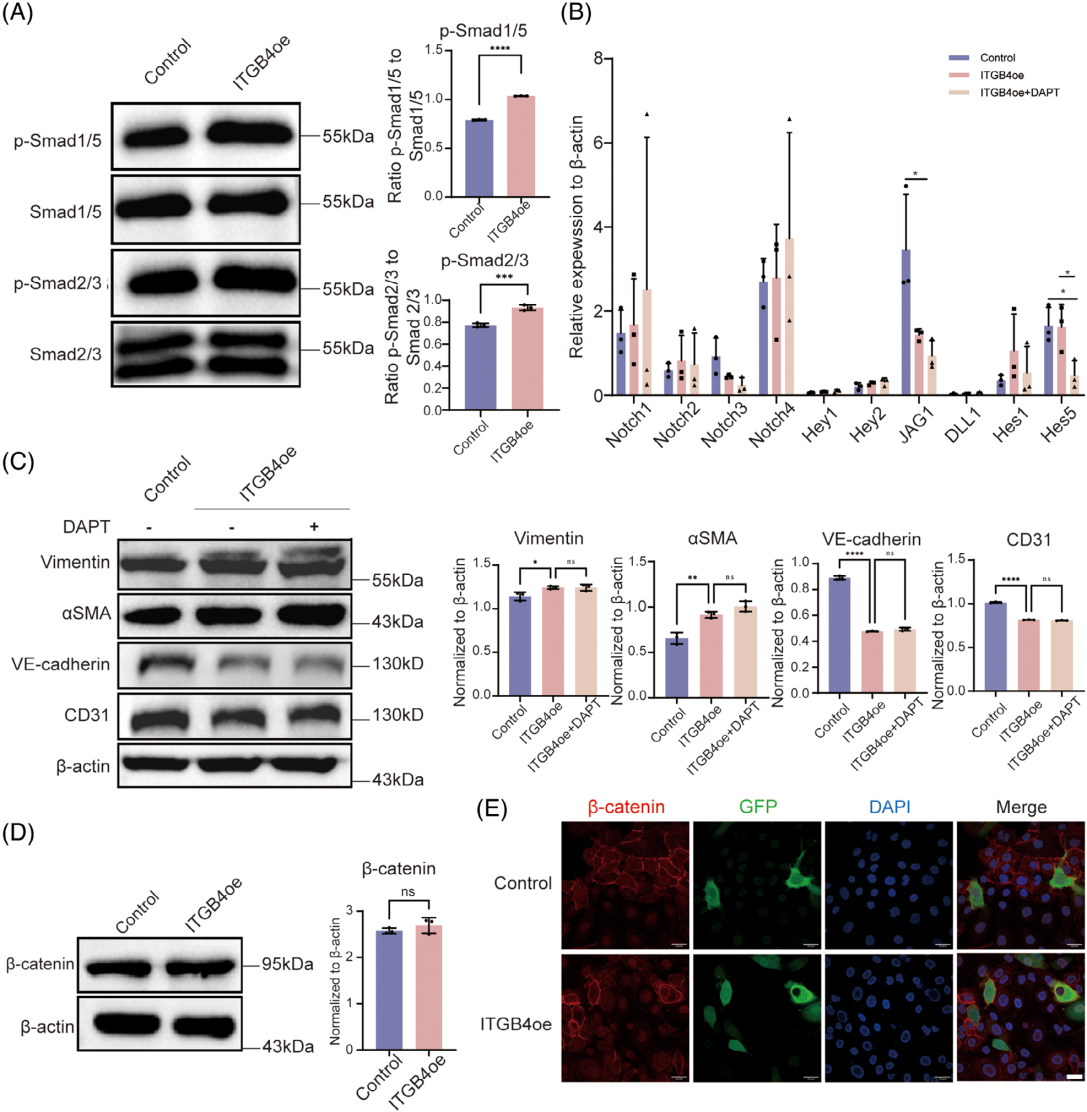
The activation of TGF‐β/BMP, rather than Wnt or Notch signaling, contributes to the ITGB4‐induced EndMT. (A) Western blot assays (left) and histograms (right) show significantly increased phosphorylation of Smad1/5 and Smad2/3. (B) RT‐qPCR analysis of Notch receptor (Notch1‐4), Notch ligand (JAG1, DLL1), and Notch target gene (Hey1, Hey2, Hes1, Hes5) expression in HBMECs overexpressing ITGB4. (C) Western blot assays (left) and histograms (right) show that treatment with Notch inhibitor DAPT could not reverse the EndMT induced by ITGB4 overexpression. (D) Total protein level of β‐catenin is not changed significantly, as shown by western blot (left) and histogram (right). (E) Representative images of immunofluorescence staining for β‐catenin showing no change in the nuclear translocation. Each image represents three replicates. Scale bar, 10 µm. ns, no significance. *p < 0.05, **p < 0.01, ****p < 0.0001. EndMT, endothelial–mesenchymal transition; HBMECs, human brain microvascular endothelial cells.

The EndMT was reported as regulated via several pathways, including TGFβ, BMP, Notch, and Wnt signaling. To determine the involvement of these in the ITGB4‐regulated EndMT, we determined the expression of respective pathway factors. The TGFβ superfamily, which is comprised of a large number of polypeptide morphogenetic factors, including TGFβ itself and BMP, mediates downstream signal transduction through Smad proteins. The binding of TGFβ to TGFβRII will recruit TGFβRI, whereafter the activated TGFβRI will phosphorylate its downstream targets Smad2/3. BMP ligands form tetrameric complexes with receptors, which in turn phosphorylate downstream Smad1/5/8 via activated type I receptors.^36^ Under ITGB4 overexpression, Smad2/3 and Smad1/5 phosphorylation were both significantly upregulated (Figure 3A), indicating that the MAPK–ERK–TGF‐β/BMP pathway was regulated downstream of ITGB4.

To examine the effect of ITGB4 on Notch signaling, we performed RT‐qPCR analysis of Notch family members (Notch 1, Notch 2, Notch 3, and Notch 4), Notch ligands (JAG1 and DLL1), and target genes (Hes1, Hes5, Hey1, Hey2). No significant changes were observed under ITGB4, with a decrease in JAG1 and Hes5 noted upon treatment with Notch inhibitor DAPT, indicating that the Notch pathway factors and target genes were not affected by ITGB4 overexpression (*p* > 0.05; Figure 3B). Further, treatment of ITGB4‐transfected HBMECs with specific Notch inhibitor DAPT did not alter the protein levels of EndMT markers (CD31, VE‐cadherin, Vimentin, and αSMA), supporting the notion that Notch signaling is not associated with the ITGB4‐induced EndMT (Figure 3C). In the context of Wnt signaling, no change in β‐catenin protein levels was noted under ITGB4 treatment (Figure 3D). Furthermore, the nuclear translocation of β‐catenin also was not affected (Figure 3E). In summary, the TGFβ/BMP pathway, rather than the Notch or Wnt signaling pathways, plays a major role in the ITGB4‐induced EndMT.

### 2.4 Atorvastatin alleviates the ITGB4‐induced EndMT by inhibiting Smad1/5 phosphorylation and promoting Smad4 ubiquitination

**FIGURE 4:**
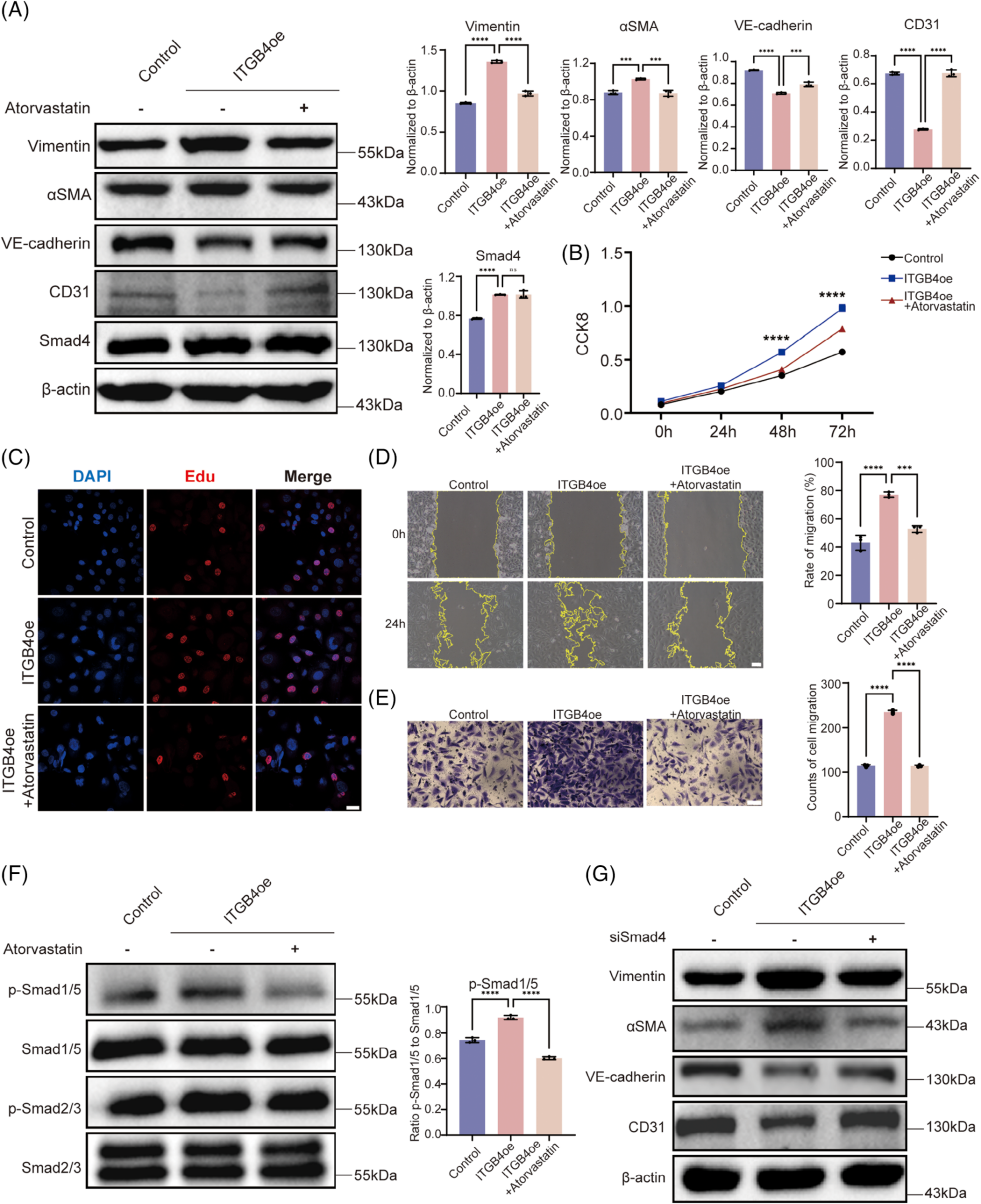
Atorvastatin alleviates the ITGB4‐induced EndMT through Smad signaling. (A) Western blot assays (left) and histograms (right) show endothelial marker (CD31 and VE‐cadherin) expression was significantly restored while that of mesenchymal markers (Vimentin and αSMA) was suppressed after treatment with atorvastatin. (B) Proliferation ability of ITGB4‐transfected and atorvastatin‐treated HBMECs, assessed via CCK8 assays. (C) The proliferation ability of HBMECs treated with atorvastatin was significantly decreased, as determined via EdU assays. Each image represents three replicates. Scale bar, 10 µm. (D) Representative images (left) and histogram (right) of the migration ability of HBMECs, as determined via wound healing assays. Each image represents three replicates. Scale bar, 100 µm. (E) Representative images (left) and histogram (right) of HBMEC migration, as determined by transwell assays. Each image represents three replicates. Scale bar in black, 25 µm. Western blot analysis of (F) phosphorylated Smad1/5 and Smad2/3 in ITGB4‐transfected HBMECs treated with atorvastatin. (G) Significantly downregulated mesenchymal markers (Vimentin and αSMA) and upregulated endothelial markers (VE‐cadherin and CD31) after Smad4 knockdown. ns, no significance. ***p < 0.001, ****p < 0.0001. EndMT, endothelial–mesenchymal transition; HBMECs, human brain microvascular endothelial cells.

Atorvastatin, an HMG‐CoA reductase inhibitor, was reported as effective in suppressing TGFβ signaling.^37^ Herein, treatment with atorvastatin significantly restored endothelial marker (CD31 and VE‐cadherin) expression while suppressing that of mesenchymal markers (Vimentin and αSMA) (Figure 4A). Further, atorvastatin suppressed the proliferative ability of ITGB4‐overexpressing HBMECs as well as their migration (Figures 4B–E and S5).

**FIGURE 5:**
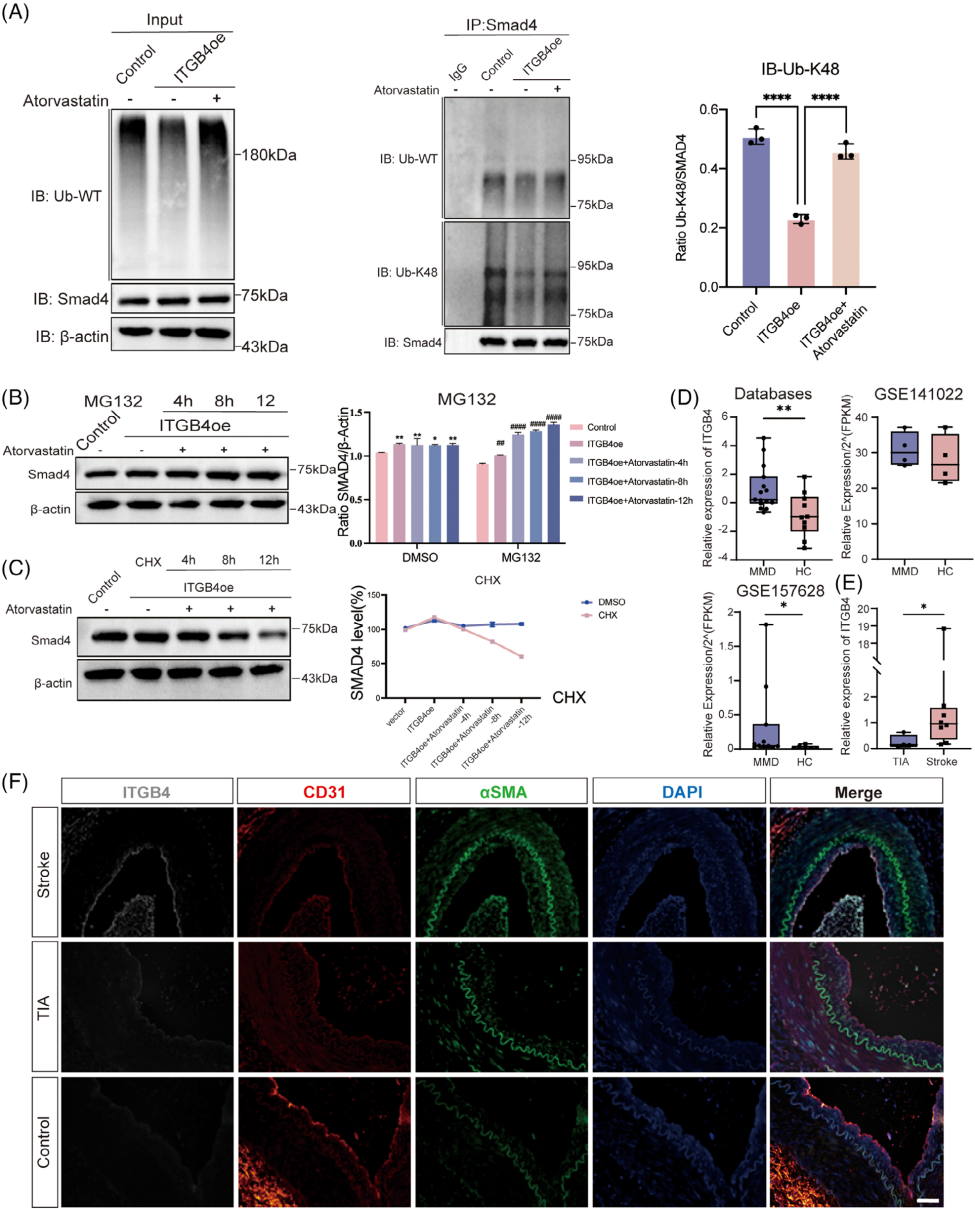
Atorvastatin promotes Smad4 ubiquitination in HBMECs, and ITGB4 is upregulated in MMD. (A) Immunoprecipitation of Smad4 wild‐type and K48‐dependent ubiquitination after atorvastatin treatment. Results from one representative experiment out of three are shown. The total protein level of Smad4 assessed via western blot are shown in HBMECs subjected to treatment with (B) proteasome inhibitor MG132 or (C) endoplasmic reticulum inhibitor CHX for 4, 8, and 12 h. (D) ITGB4 mRNA expression in public databases (GSE141022, GSE157628). (E) ITGB4 mRNA expression is significantly upregulated in MMD patients with stroke manifestation. (F) Immunofluorescence staining of ITGB4, CD31, and αSMA in the superficial temporal artery of patients with MMD. *p < 0.05, **p < 0.01. HBMECs, human brain microvascular endothelial cells; MMD, moyamoya disease.

To further explore the mechanism through which atorvastatin inhibits the ITGB4‐induced EndMT, we examined Smad2/3 and Smad1/5 phosphorylation levels via western blotting (Figure 4F). Smad1/5 phosphorylation was completely reversed, while Smad2/3 phosphorylation decreased slightly, but significantly (*p* < 0.05; Figure S6). This indicated that another mechanism may be involved in EndMT reversal. Smad4 was reported as the only common mediator of Smad signaling, with both phosphorylated Smad2/3 and Smad1/5 translocating into the nucleus after forming a complex with Smad4, indicative of a broad impact on both TGFβ and BMP pathways.^38^ First, we utilized Smad4 siRNA to examine its regulatory role in the EndMT. After Smad4 knockdown, CD31 and VE‐cadherin protein levels were significantly enhanced, while those of Vimentin and αSMA were significantly downregulated (*p* < 0.05; Figures 4G and S7). These findings suggest that Smad4 knockdown could inhibit the EndMT induced by ITGB4. The activity and stability of Smad4 can be regulated via posttranslational modifications such as SUMOylation, ubiquitination, and phosphorylation.^39^, ^40^ Recent studies have shown that K48‐dependent polyubiquitination may be involved in MMD.^41^ We performed coimmunoprecipitation assays to assess the effect of atorvastatin on Smad4 polyubiquitination. Our results confirmed that Smad4 is ubiquitinated and that ITGB4 can inhibit Smad4 protein degradation by impeding its ubiquitination. Following atorvastatin treatment, Smad4 ubiquitination was upregulated, accelerating its degradation (Figure 5A). Furthermore, K48‐dependent polyubiquitination was consistently reversed after treatment with atorvastatin (Figure 5A). MG132 is a proteasome inhibitor that suppresses the degradation of ubiquitinated proteins. We added MG132 to HBMECs treated with atorvastatin for 4, 8, and 12 h. Overexpression of ITGB4 upregulated Smad4, with Smad4 degradation being significantly suppressed following atorvastatin and MG132 treatment, in a time‐dependent manner (*p* < 0.05; Figure 5B). We also used the endoplasmic reticulum inhibitor CHX to inhibit protein synthesis and observe Smad4 degradation. Compared with the control group, addition of atorvastatin and CHX gradually decreased Smad4 expression in a time‐dependent manner, with the half‐life of Smad4 protein becoming shorter (*p* < 0.05; Figure 5C).

### 2.5 ITGB4 is significantly upregulated in the arterial endothelium during the EndMT in MMD

**FIGURE 6:**
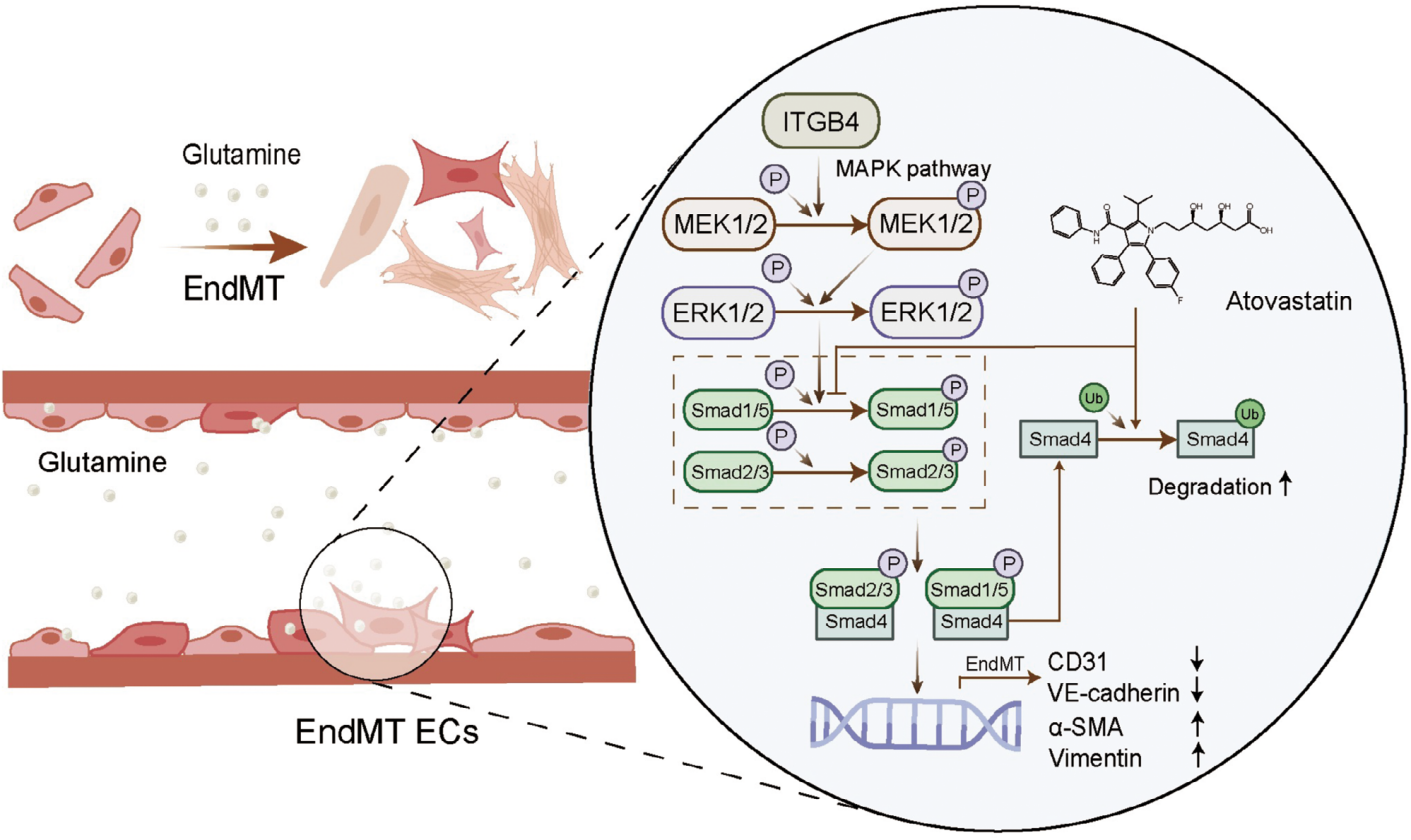
Diagram of the study is summarized. EndMT, endothelial‐to‐mesenchymal transition; EC, endothelial cell.

To determine the expression of ITGB4 in MMD patients, we used publicly available databases (GEO141022 and GEO157628) (Table S4). In the GEO157628 dataset, ITGB4 was significantly increased in 11 patients with MMD compared with six patients with aneurysm as controls (Figure 5D). A similar increase in the mRNA level of ITGB4 was noted in GEO141022, although no significant difference was observed owing to the limited number of enrolled patients (four MMD patients and four aneurysm patients; Figure 5D). We performed RT‐qPCR on 12 bulk superficial temporal artery samples from patients with MMD, including four with the TIA subtype and eight with the stroke subtype. ITGB4 was significantly increased in MMD patients with stroke (*p* < 0.05; Figure 5E). We detected the protein level of ITGB4 using immunofluorescence staining and found higher ITGB4 levels in patients with stroke (Figure 5F). We also observed a significant upregulation of mesenchymal marker αSMA, which confirmed the EndMT in MMD (Figure 5F). These results suggest that EndMT occurs during MMD pathogenesis and ITGB4 represents a potential biomarker of stroke risk (Figure 6).

## 3 DISCUSSION

In the present study, we suggest that glutamine induces the EndMT via ITGB4 and the MAPK–ERK/Smad pathway, being associated with an independently increased risk of stroke in patients with MMD. Furthermore, we showed that atorvastatin inhibits the ITGB4‐induced EndMT through the suppression of Smad1/5 phosphorylation and Smad4 K48‐dependent ubiquitination. Our study implies that high glutamine levels may be an independent risk factor for intracerebral hemorrhage or infarction in patients with MMD. Further, ITGB4 represents a potential biomarker reflecting stroke risk in patients with MMD. Finally, we highlighted atorvastatin as a promising treatment candidate for suppressing the ITGB4‐induced EndMT in MMD.

Glutamine, as the most abundant amino acid in the human body, is essential for a variety of cellular processes, including energy production, as it replenishes the pool of tricarboxylic acid cycle intermediates.^42^ Recent studies have shown that glutamine contributes to multiple pathological processes, such as cardiac hypertrophy, liver disease, and tumor progression.^43^, ^44^, ^45^, ^46^, ^47^, ^48^ In this study, we found that higher glutamine levels were associated with stroke in patients with MMD. We also recruited 59 healthy volunteers and found that serum glutamine levels were significantly higher in patients (*p* = 0.016; Mann–Whitney test). However, the results were not adjusted for age, sex, or other demographic characteristics, which requires further research. Some studies have also suggested higher phenylacetylglutamine levels in recurrent stroke patients.^49^ The association between glutamine levels and stroke in healthy individuals remains to be further interrogated. Glutamine has various effects on the body, providing many essential nitrogen sources and participating in glutathione synthesis. While we believe that glutamine levels represent a risk factor for stroke in patients with MMD, the reduction of glutamine levels may not be a favorable goal. Previous in vitro experiments have revealed the role of glutamine in regulating the proliferation and differentiation of mesenchymal stem cells, immune cells, and vascular smooth muscle cells.^50^, ^51^, ^52^, ^53^ We found that, in endothelial cells, glutamine was not only associated with proliferation ability but also promoted the EndMT. The EndMT has been reported to play a central role in cardiovascular and cerebrovascular diseases, with EndMT reversal representing a potential therapeutic approach for patients with MMD. The upregulation of ITGB4 after treatment with glutamine could be attributed to several factors. Glutamine is involved in protein synthesis, and ITGB4 upregulation may result from increased translation or the stabilization of ITGB4 mRNA and protein. Glutamine enhances amino acid availability and promotes protein production, leading to higher ITGB4 levels. Glutamine can also activate various signaling pathways, including those involved in cell adhesion and migration, thus directly regulating ITGB4 expression.^54^, ^55^, ^56^ ITGB4 is an integrin subunit that plays a crucial role in cell adhesion to the ECM.^57^ Glutamine‐induced ITGB4 upregulation may underpin a cellular response that enhances cell adhesion and migration in order to facilitate the repair and regeneration of endothelial cells. However, further experiments are required to confirm the specific mechanism underlying ITGB4 upregulation in glutamine‐treated HBMECs.

To date, the diagnosis of MMD has largely relied on radiological findings, and its pathogenesis has remained elusive. Although previous studies have reported smooth muscle cell proliferation observed during MMD autopsy,^58^ recent evidence has emphasized the importance of endothelial barrier function in the development of MMD.^59^, ^60^, ^61^ Elevated levels of the mesenchymal marker MMP‐9 were also reported in patient serum and the endothelial‐matrix interface of vessel specimens.^62^, ^63^ Studies have suggested that endothelial barrier dysfunction caused by the EndMT can contribute to infarction or hemorrhagic diseases.^31^, ^32^, ^64^, ^65^ We performed immunofluorescence staining of the superficial temporal artery and observed a consistent EndMT phenomenon with elevated ITGB4 expression, which provides evidence for endothelial changes during MMD pathogenesis. TGFβ, BMP, Notch, and Wnt signaling have been reported to independently regulate the EndMT process.^66^ In this study, we observed that ITGB4 induces the EndMT through the MAPK–ERK–TGF‐β/BMP axis, which could be reversed via Smad4 knockdown.

Owing to the unknown etiology and lack of animal models of MMD, no curative treatment is currently available, with symptomatic therapy for stroke manifestations constituting the main treatment regimen. That is, no drugs or revascularization approaches can effectively control or reverse MMD pathogenesis. Instead, drugs are mainly used for symptomatic and supportive treatment or perioperative management.^11^ Atorvastatin is a widely used lipid‐lowering drug reported to regulate TGFβ signaling.^67^ Herein, treatment with atorvastatin significantly reversed the ITGB4‐induced EndMT. Moreover, atorvastatin inhibited Smad1/5 phosphorylation and promoted the K48‐dependent ubiquitination of Smad4 to alleviate ITGB4‐induced EndMT. These results suggest that atorvastatin holds potential as a therapeutic for the prevention of MMD progression. Interestingly, the RNF213 mutation, which is found in 30% of patients with MMD, was reported to disrupt this K48‐dependent poly‐ubiquitylation event.^41^ Patients with different genotypes may therefore respond differently to atorvastatin treatment. Considering the complex role of RNF213 in MMD, future studies are needed to clarify optimal treatment modalities.

The present study has several limitations. Since it is a prospective cohort study, there may have been observer bias. In addition, the observation time of this study was relatively long, yet we could not analyze patient prognosis. While we found that glutamine levels were associated with stroke in patients with MMD, whether glutamine is associated with stroke in healthy individuals remains to be explored in future cohort studies. Finally, we acknowledge that at present, there is no established animal model that fully replicates MMD. Although RNF213 has been investigated, they fail to detect stenosis or occlusion of the intracranial arteries in Rnf213^+/+^ mice or Rnf213^−/−^ mice.^68^, ^69^ Due to the current limitations in modeling MMD in animals, our conclusions are therefore derived from the available in vitro data and supported by clinical observations. We believe that our study's findings contribute valuable insights into the disease's pathogenesis and could be foundational for future research as more advanced models become available.

## 4 MATERIALS AND METHODS

### 4.1 Study participants

A total of 500 patients diagnosed with MMD were admitted to the Department of Neurosurgery, Beijing Tiantan Hospital from September 2020 to December 2022, becoming part of the MOYAOMICS cohort (ChiCTR.org; ChiCTR2200061889). The inclusion criteria were as follows: (1) patients with MMD diagnosed via DSA or MRA; (2) patients or their guardians agreed to participate in the study; and (3) patients completed the relevant examinations. Healthy controls who underwent physical examinations in the hospital during the same period were also included. The exclusion criteria were as follows: (1) history of cardiac, renal, or systemic vascular disease; (2) patients with coagulation dysfunction. We excluded 82 pediatric patients, 11 patients aged > 60 years, and 47 patients without glutamine data. A total of 360 patients were prospectively enrolled in this study. The guidelines of the Research Committee on Spontaneous Occlusion of the Circle of Willis were used to diagnose MMD using DSA.^70^ This study was approved by the Ethics Committee of Beijing Tiantan Hospital, Capital Medical University (No. 2021YFC2500502), and informed consent was obtained from all participants.

### 4.2 Data collection

Demographic data, medical histories, clinical features, laboratory examinations, and radiological examinations were extracted from the electronic medical record system. Age and sex were included in demographic data analyses. Medical history included hypertension, diabetes mellitus, hyperlipidemia, cigarette smoking, and alcohol consumption. The clinical features included heart rate, SBP, DBP, and BMI. Laboratory examinations included RBC count, HGB count, HCT value, PLT count, creatinine, uric acid, TG, TC, HDL‐C, LDL‐C, ApoA_1_, ApoB, and Hcy. Serum Hcy exceeding 15 µmol/L was diagnosed as HHcy, consistent with our previous studies.^2^, ^71^

### 4.3 Blood sample preparation and extraction

After the participants had been admitted for over 12 h, their blood samples were collected in the morning and stored at −80°C. Before extraction, all samples were thawed and vortexed for 10 s. Fifty microliters of each was transferred to a centrifuge tube and mixed with 250 µL of 20% acetonitrile/methanol. The sample was then vortexed for 3 min, centrifuged at 13,800 xg for 10 min at 4°C, and 250 µL of supernatant was transferred into a new centrifuge tube. The supernatants were stored in a −20°C refrigerator for 30 min and centrifuged at 13,800 xg for 10 min at 4°C. After centrifugation, 180 µL of the supernatant was transferred through a Protein Precipitation Plate for further LC–MS analysis.

### 4.4 Ultra‐performance liquid chromatography and MS/MS

The sample extracts were analyzed using an LC–ESI–MS/MS system (ultra‐performance liquid chromatography [UPLC]; ExionLC AD; AB Sciex, Canada; MS, QTRAP® 6500+ System; AB Sciex). UPLC conditions were as follows: solvent system, water with 2 mM ammonium acetate and 0.04% formic acid (A), acetonitrile with 2 mM ammonium acetate and 0.04% formic acid (B). The gradient was started at 90% B (0–1.2 min), decreased to 60% B (9 min), 40% B (10–11 min), finally ramped back to 90% B (11.01–15 min); flow rate, 0.4 mL/min; temperature, 40°C; injection volume: 2 µL. The AB 6500+ QTRAP LC–MS/MS System, equipped with an ESI Turbo Ion‐Spray interface, operated in both positive and negative ion modes, was controlled via Analyst 1.6 software (AB Sciex).

### 4.5 Culture and treatment of HBMECs

Commercially available HBMECs (#1000; ScienCell, USA) were cultured in endothelial cell medium (#1001; ScienCell) with 5% FBS according to the manufacturer's guidelines and were not used beyond passage 10. The HBMECs were cultured and maintained in a humidified atmosphere at 37°C and 5% CO_2_. U0126 (S1102; Selleck, USA), DAPT (S2215, Selleck), and atorvastatin (S5715; Selleck) were dissolved with dimethyl sulfoxide. HBMECs were cultured with glutamine at a concentration of 20 mM, U0126 at a concentration of 1:1000, or atorvastatin at a concentration of 1:1000 before subsequent experiments.

### 4.6 RNA‐sequencing

TRIzol (#15596‐018; Ambion, USA) was used to obtain total RNA from the samples for RNA‐seq. A Qubit® 2.0 Fluorometer (Life Technologies, USA) was used to measure RNA purity and concentration. RNA integrity was assessed using an RNA Nano 6000 Assay Kit and a Bioanalyzer 2100 system (Agilent Technologies, USA). RNA‐seq was performed according to a previous method.^72^ Differentially expressed genes were determined based on a fold change >1.20 or <0.83, with *p* < 0.05.

### 4.7 Real time‐quantitative polymerase chain reaction

After the different treatments, total RNA was extracted from HBMECs using TRIzol (#15596‐018; Ambion). cDNA was then reverse‐transcribed using a PrimeScrip RT Reagent Kit (RR047; Takara, Japan). RT‐qPCR was performed using TB Green Premix Ex Taq (RR820; Takara) on a QuantStudio™ 3 System (Applied Biosystems, Thermo Scientific, USA). The specific primers are described in Table S5. Relative expression was determined via the ΔΔCt method, with β‐actin as the reference gene.

### 4.8 siRNA and adenovirus transfection

At the appropriate time points, HBMECs were transfected with siRNAs using Lipofectamine 3000 Transfection Reagent (L3000001; Invitrogen, USA) in order to knockdown ITGB4 gene expression. Following 4–6 h of transfection, the medium was replaced, and the cells were harvested for subsequent experiments after 48 h. Adenoviral vectors were used for transient overexpression of target genes. HUVECs were infected with an adenovirus encoding ITGB4 at a multiplicity of infection of 20, and the infected HBMECs were harvested after 72 h. The ITGB4 siRNA sequence is shown in Table S6.

### 4.9 Proliferation assays

For CCK8 assays, 5000 cells were seeded in each well of a 96‐well plate (#3599; Corning, USA). Then, 10 µL of CellTiter‐Blue cell viability reagent (#G8080; Promega, USA) was added, and the optical density at 450 nm was measured. For the EdU assay, an appropriate number of HBMECs were seeded in six‐well plates (#3516; Corning), and 10 µM EdU was added to the medium for 4 h. The cells were fixed with 4% paraformaldehyde for 15 min. Cells were washed with Dulbecco's phosphate‐buffered saline (DPBS; Sigma, USA) for 3–5 min each and tested using an EdU kit (C0078; Beyotime, China), according to the manufacturer's instructions.

### 4.10 Migration assay

After HBMECs were treated, a straight scratch line was made from the top to the bottom of the well using a yellow tip (T‐200; Axygen, USA). The scratch was observed at appropriate times and analyzed using ImageJ software (V1.8.0; NIH, USA).

### 4.11 Western blot assay

Protein samples were collected using radioimmunoprecipitation assay (RIPA) lysis buffer (Sigma) for 30 min on ice and quantified using a bicinchoninic acid protein assay kit (KeyGen, China). Equivalent amounts of cell lysate were separated using a precast gel (Solarbio, China) and transferred onto polyvinylidene fluoride membranes (Millipore, USA). The membranes were then blocked for 1 h at room temperature and immunoblotted at 4°C with primary antibodies overnight. The next day, membranes were washed with Tris‐Buffered Saline and Tween (TBST) at least 3 times and then incubated with appropriate secondary antibodies (Abmart, China). Bands were visualized using the Immobilon Western Chemiluminescent HRP Substrate (Millipore, Billerica, MA, USA). The antibodies used are listed in Table S7.

### 4.12 Immunofluorescence

After the HBMECs were cultured, and cell viability reached 90%, they were added to a 12‐well plate at a density of 8 × 10^4^ cells per well. HBMECs were then treated and harvested. After the medium was removed, the HBMECs were washed three times with DPBS and fixed with 4% paraformaldehyde for 10 min. Thereafter, 400 µL of TBST containing 0.5% TritonX‐100 was added to permeabilize the cells and incubated at room temperature for 10 min. The solution was aspirated and washed three times with TBST containing 3% Bovine Serum Albumin (BSA). Then, 400 µL sheep serum (dilution ratio 1:10) was added to block samples, which were kept at room temperature for 2 h. Primary antibodies were added and incubated overnight at 4°C. After three washes with TBST on the following day, corresponding immunofluorescent secondary antibodies were added and incubated for 2 h on a shaker in the dark. The secondary antibody was aspirated, and DAPI was added for nuclear staining.

### 4.13 Coimmunoprecipitation assay

After HBMECs were treated, the corresponding volume of MG132 was added 6–8 h before collecting total protein using RIPA. We prepared isotype controls by adding 700 µL lysate, 80 µL agarose Protein A/G Beads, and 2 µg IgG Isotype to a 1.5 mL imported EP tube and incubating for 30 min at 4°C with a rotary mixer. We then centrifuged at 13,800 xg and 4°C for 15 min to precipitate unlysed impurities and preliminarily purify the protein. After centrifugation, 150 µL supernatant was prepared and transferred to the washed beads, followed by incubation for 30 min at 4°C in a rotary mixer. A pipette was used to transfer the supernatant to a new 1.5 mL EP tube, whereafter it was placed in an ice box. The remaining precipitation steps were the same as those described above, and preheated loading buffer was added. We added 2 µL anti‐Smad4 to the supernatant, incubated overnight in a 4°C refrigerator rotary mixer, then adding 80 µL protein A/G Beads and continued to rotate and incubate at 4°C in the refrigerator rotary mixer for 3 h. Thereafter, we added 80 µL loading buffer and heated at 100°C for 10 min, then centrifuging at 4°C. The sample was stored at −20°C together with input and IgG isotypes.

### 4.14 Statistical analysis

SPSS (version 25.0, IBM, USA), Prism (version 8.00, GraphPad Software, USA), and R (version 4.0.5, USA) were used for statistical analyses. The Kolmogorov–Smirnov test was used to confirm whether the data conformed to normality. Continuous variables with a normal distribution are presented as the mean ± standard deviation (SD) and were tested using a t‐test or ANOVA. Otherwise, these were presented as a median (IQR) and tested using the Mann–Whitney or Kruskal–Wallis test. Categorical variables were tested using the chi‐squared test or Linear‐by‐Linear Association. A two‐tailed probability value of less than 0.05 was considered to indicate statistical significance.

## AUTHOR CONTRIBUTIONS

P. G., Q. H., and J. Z. designed the study. P. G. and Q. H. wrote the manuscript and performed the statistical analyses. Q. H., C. L., Z. Z., and S. M. performed the experiments. J. L. and C. T. performed bioinformatics analysis. C. Z., W. L., X. Y., Y. Z., J. W., Q. Z., Y. Z., and B. Z. collected data and patient samples. Z. Z. illustrates the graphical abstract. D. Z. and J. Z. supervised the study and reviewed the manuscript. All authors have read and approved the final manuscript.

## CONFLICT OF INTEREST STATEMENT

The authors declared no conflict of interest.

## ETHICS STATEMENT AND CONSENT TO PARTICIPATE

This study was approved by the Ethics Committee of Beijing Tiantan Hospital, Capital Medical University (No. 2021YFC2500502), the study was registered in Chinese Clinical Trial Registry (ChiCTR2200061889) and informed consent was obtained from all participants.

## Supporting information

Supporting Information

## Data Availability

The raw data of this study are available from the Gene Expression Omnibus (GEO) with accession number GSE259423 (https://www.ncbi.nlm.nih.gov/geo/query/acc.cgi?acc=GSE5259423) upon reasonable request. The datasets analyzed in this study were downloaded from the GEO database with accession numbers GSE157628 (https://www.ncbi.nlm.nih.gov/geo/query/acc.cgi?acc=GSE157628) and GSE141022 (https://www.ncbi.nlm.nih.gov/geo/query/acc.cgi?acc=GSE141022).
