# Supplementary material for: High glutamine increases stroke risk by inducing the endothelial‐to‐mesenchymal transition in moyamoya disease
Source: MedComm (2020). 2024 Apr 15;5(5):e525. doi: 10.1002/mco2.525 (PMC11018113; doi:10.1002/mco2.525)
Supplement: Supplementary file 1 — Supporting Information [file MCO2-5-e525-s001.docx]

***Supplementary Materials***

**High glutamine increases stroke risk by inducing the endothelial-to-mesenchymal transition in moyamoya disease**

Running title: High glutamine induces EndMT in MMD

Qiheng He, M.D.^1^, Junsheng Li, M.D.^1^, Chuming Tao, M.D.^1^, Chaofan Zeng, M.D.^1^, Chenglong Liu, M.D.^1^, Zhiyao Zheng, M.D.^1, 4, 5^, Siqi Mou, M.D.^1^, Wei Liu, M.D.^1^, Bojian Zhang, M.D.^1^, Xiaofan Yu, M.D.^1^, Yuanren Zhai, M.D.^1^, Jia Wang, M.D.^1, 3^, Qian Zhang, M.D.^1^, Yan Zhang, Ph.D.^1^, Dong Zhang, Ph.D.^2^*, Jizong Zhao, Ph.D.^1, 3^*, Peicong Ge, Ph.D.^1^*

Running Title: Glutamine induce EndMT in MMD

Affiliations:

^1^ Department of Neurosurgery, Beijing Tiantan Hospital, Capital Medical University, Beijing 100070, China

^2^ Department of Neurosurgery, Beijing Hospital, Beijing 100005, China

^3^ China National Clinical Research Center for Neurological Diseases, Beijing 100070, China

^4^ Research Unit of Accurate Diagnosis, Treatment, and Translational Medicine of Brain Tumors, Chinese Academy of Medical Sciences and Peking Union Medical College, Beijing 100730, China

^5^ Department of Neurosurgery, Peking Union Medical College Hospital, Chinese Academy of Medical Sciences and Peking Union Medical College, Beijing 100730, China

**Corresponding author:**

Jizong Zhao, Email: [zhaojizong@bjtth.org](mailto:zhaojizong@bjtth.org)

Dong Zhang, Email: [zhangdong0660@aliyun.com](mailto:zhangdong0660@aliyun.com)

Peicong Ge, Email: [gepeicong@163.com](mailto:gepeicong@163.com)

**Supplementary Tables**

| **Table S1.** Subgroup characteristics of MMD patients. | | | | |
| --- | --- | --- | --- | --- |
| Variables | All patients (N = 360) | TIA (n=114) | Stroke | |
|  |  |  | Infarction (n=145) | Hemorrhage (n=101) |
| Age, y, median (IQR) | 43.00 (34.00 - 50.00) | 41.50 (34.00 - 48.00) | 43.00 (35.00 - 50.50) | 43.00 (33.50 - 50.00) |
| Sex, n (%) |  |  |  |  |
| Male | 150 (41.67) | 47 (31.33) | 69 (46.00) | 34 (22.67) |
| Female | 210 (58.33) | 67 (31.90) | 76 (36.19) | 67 (31.90) |
| History of risk factors, n (%) |  |  |  |  |
| Hypertension | 131 (36.39) | 35 (26.72) | 67 (51.15) | 29 (22.1) |
| Diabetes mellitus | 59 (16.39) | 16 (27.12) | 39 (66.10) | 4 (6.78) |
| Hyperlipidemia | 54 (15.00) | 17 (31.48) | 28 (51.85) | 9 (16.67) |
| Cigarette smoking | 71 (19.72) | 20 (28.17) | 34 (47.89) | 17 (23.94) |
| Alcohol drinking | 42 (11.67) | 14 (33.33) | 20 (47.62) | 8 (19.05) |
| Clinical features, median (IQR) |  |  |  |  |
| Heart rate, bpm | 78.00 (75.00 - 80.00) | 78.00 (75.75 - 80.00) | 78.00 (74.00 - 80.00) | 79.00 (76.00 - 82.00) |
| SBP, mmHg | 132.00 (124.00 - 140.00) | 133.00 (124.00 - 140.00) | 135.00 (125.50 - 140.00) | 130.00 (121.50 - 138.00) |
| DBP, mmHg | 81.00 (76.00 - 89.00) | 80.00 (75.75 - 89.25) | 82.00 (77.00 - 90.00) | 80.00 (74.00 - 88.00) |
| BMI, kg/m^2^ | 25.00 (22.49 - 27.78) | 25.33 (22.77 - 28.09) | 25.61 (23.17 - 28.24) | 24.03 (21.92 - 26.20) |
| Laboratory results |  |  |  |  |
| RBC, 10^12^/L, mean ± SD | 4.65 ± 0.51 | 4.69 ± 0.51 | 4.66 ± 0.53 | 4.58 ± 0.47 |
| HGB, g/L, mean ± SD | 140.44 ±18.32 | 143.04 ± 16.49 | 141.06 ± 19.77 | 136.61 ± 17.68 |
| HCT, L/L, median (IQR) | 0.41 (0.38 - 0.45) | 0.41 (0.39 - 0.45) | 0.41 (0.38 - 0.46) | 0.41 (0.38 - 0.43) |
| PLT count, 10^9^/L, median (IQR) | 248.00 (209.50 - 284.75) | 249.50 (210.25 - 282.25) | 250.00 (218.00 - 297.50) | 244.00 (204.00 - 279.50) |
| Creatinine, μmol/L, median (IQR) | 54.75 (46.33 - 66.98) | 53.95 (46.63 - 66.68) | 57.80 (47.15 - 67.95) | 53.10 (45.20 - 66.95) |
| Uric acid, μmol/L, median (IQR) | 4.50 (3.80 - 5.60) | 4.50 (3.80 - 5.73) | 4.50 (3.70 - 5.55) | 4.60 (3.70 - 5.65) |
| TG, mmol/L, median (IQR) | 1.20 (0.82 - 1.64) | 1.24 (0.84 - 1.75) | 1.20 (0.84 - 1.59) | 1.13 (0.79 - 1.64) |
| TC, mmol/L, median (IQR) | 4.23 (3.55 - 4.83) | 4.23 (3.49 - 4.89) | 3.93 (3.34 - 4.60) | 4.35 (3.90 - 5.03) |
| HDL-C, mmol/L, median (IQR) | 1.30 (1.12 - 1.49) | 1.31 (1.09 - 1.51) | 1.25 (1.10 - 1.41) | 1.34 (1.17 - 1.52) |
| LDL-C, mmol/L, median (IQR) | 2.40 (1.84 - 2.98) | 2.36 (1.79 - 2.99) | 2.15 (1.70 - 2.74) | 2.55 (2.19 - 3.26) |
| ApoA_1_, g/L, median (IQR) | 1.29 (1.15 - 1.45) | 1.32 (1.15 - 1.46) | 1.25 (1.11 - 1.44) | 1.30 (1.17 - 1.46) |
| ApoB, g/L, median (IQR) | 0.82 (0.70 - 0.97) | 0.84 (0.69 - 0.96) | 0.81 (0.67 - 0.95) | 0.82 (0.71 - 1.02) |
| Hcy, μmol/L, median (IQR) | 12.00 (9.30 - 15.08) | 11.11 (8.59 - 15.45) | 12.29 (9.65 - 15.77) | 11.90 (9.38 - 14.35) |
| HHcy, n (%) | 91 (25.28) | 29 (31.87) | 40 (43.96) | 22 (24.18) |
| Glutamine, 10^3μmol/L, median (IQR) | 3.58 (3.24 - 3.94) | 3.51 (3.16 - 3.71) | 3.68 (3.27 - 4.06) | 3.60 (3.28 - 3.98) |
| MMD, moyamoya disease; SD, standard deviation; TIA, transient ischemic attack; mRS, modified Rankin Scale; SBP, systolic blood pressure; DBP, diastolic blood pressure; BMI, body mass index; UA, uric acid; TG, triglyceride; TC, total cholesterol; HDL-C, high-density lipoprotein cholesterol; LDL-C, low-density lipoprotein cholesterol; ApoA_1_, apolipoprotein A_1_; ApoB, apolipoprotein B; Hcy, homocysteine; HHcy, hyperhomocysteinemia. *P < 0.05, significant difference | | | | |

| **Table S2. Characteristics of MMD patients according to glutamine tertiles** | | | | |
| --- | --- | --- | --- | --- |
| Variables | Glutamine Tertiles | | | |
|  | T1 (N = 120) | T2 (N = 120) | T3 (N = 120) | *P Trend* |
| Age, y, median (IQR) | 42.00 (34.00 - 48.00) | 43.00 (34.00 - 49.00) | 44.50 (34.25 - 51.00) | 0.235 |
| Sex, n (%) |  |  |  | <0.001 |
| Male | 30 (20.00) | 46 (30.67) | 74 (49.33) |  |
| Female | 90 (42.86) | 74 (35.24) | 46 (21.90) |  |
| History of risk factors, n (%) |  |  |  |  |
| Hypertension | 42 (32.06) | 43 (32.82) | 46 (35.11) | 0.592 |
| Diabetes mellitus | 18 (30.51) | 16 (27.12) | 25 (42.37) | 0.223 |
| Hyperlipidemia | 15 (27.78) | 20 (37.04) | 19 (35.19) | 0.470 |
| Cigarette smoking | 16 (22.54) | 22 (30.99) | 33 (46.48) | 0.006 |
| Alcohol drinking | 12 (28.57) | 15 (35.71) | 15 (35.71) | 0.547 |
| Clinical features, median (IQR) |  |  |  |  |
| Heart rate, bpm | 78.0 (75.0 - 80.0) | 78.0 (70.2 - 80.0) | 78.0 (75.0 - 81.5) | 0.598 |
| SBP, mmHg | 134.0 (124.0 - 140.0) | 132.0 (123.3 - 140.0) | 132.0 (124.0 - 140.0) | 0.823 |
| DBP, mmHg | 81.0 (75.0 - 90.0) | 82.0 (77.0 - 89.0) | 80.0 (77.0 - 88.0) | 0.720 |
| BMI, kg/m^2^ | 24.6 (22.5 - 27.3) | 25.3 (22.5 - 28.0) | 25.3 (22.5 - 28.0) | 0.440 |
| Laboratory results, median ± IQR |  |  |  |  |
| Lymphocyte count, 10^9^/L, median (IQR) | 1.92 (1.60 - 2.34) | 1.95 (1.52 - 2.49) | 1.90 (1.47 - 2.42) | 0.697 |
| Neutrophil count, 10^9^/L, median (IQR) | 4.11 (3.50 - 5.04) | 4.03 (3.18 - 5.39) | 4.59 (3.48 - 5.40) | 0.244 |
| Monocyte count, 10^9^/L, median (IQR) | 0.33 (0.27 - 0.43) | 0.36 (0.28 - 0.47) | 0.36 (0.30 - 0.47) | 0.052 |
| RBC, 10^12^/L, median (IQR) | 4.55 (4.32 - 4.89) | 4.64 (4.28 - 5.04) | 4.73 (4.37 - 5.08) | 0.190 |
| HGB, g/L, median (IQR) | 137.00 (129.25 - 146.50) | 141.50 (128.25 - 153.00) | 145.00 (132.25 - 159.00) | 0.006 |
| HCT, L/L, median (IQR) | 0.41 (0.38 - 0.43) | 0.42 (0.38 - 0.44) | 0.42 (0.39 - 0.46) | 0.012 |
| PLT count, 10^9^/L, median (IQR) | 251.50 (219.25 - 287.50) | 242.50 (206.25 - 292.00) | 247.50 (200.50 - 280.75) | 0.287 |
| Creatinine, μmol/L, median (IQR) | 50.05 (45.45 - 58.85) | 55.70 (45.83 - 67.88) | 61.60 (48.88 - 71.33) | <0.001 |
| Uric acid, μmol/L, mean ± SD | 4.47 ± 1.32 | 4.82 ± 1.25 | 4.82 ± 1.51 | 0.067 |
| TG, mmol/L, median (IQR) | 1.18 (0.81 - 1.59) | 1.26 (0.84 - 1.92) | 1.15 (0.86 - 1.51) | 0.314 |
| TC, mmol/L, median (IQR) | 4.23 (3.49 - 4.83) | 4.25 (3.60 - 4.92) | 4.20 (3.45 - 4.70) | 0.482 |
| HDL-C, mmol/L, median (IQR) | 1.32 (1.15 - 1.51) | 1.27 (1.11 - 1.47) | 1.31 (1.07 - 1.47) | 0.308 |
| LDL-C, mmol/L, median (IQR) | 2.40 (1.85 - 3.04) | 2.39 (1.89 - 2.95) | 2.37 (1.80 - 2.98) | 0.913 |
| ApoA_1_, g/L, median (IQR) | 1.31 (1.19 - 1.46) | 1.29 (1.13 - 1.44) | 1.26 (1.07 - 1.42) | 0.053 |
| ApoB, g/L, median (IQR) | 0.82 (0.70 - 0.98) | 0.86 (0.70 - 0.97) | 0.82 (0.67 - 0.98) | 0.731 |
| Hcy, μmol/L, median (IQR) | 11.05 (8.80 - 13.75) | 11.69 (9.35 - 16.05) | 12.69 (9.78 - 16.35) | 0.019 |
| HHcy, n (%) | 21 (23.08) | 32 (35.16) | 38 (41.76) | 0.012 |
| MMD, moyamoya disease; SD, standard deviation; TIA, transient ischemic attack; mRS, modified Rankin Scale; SBP, systolic blood pressure; DBP, diastolic blood pressure; BMI, body mass index; WBC, white blood cell; MPV, mean platelet volume; UA, uric acid; TG, triglyceride; TC, total cholesterol; HDL-C, high-density lipoprotein cholesterol; LDL-C, low-density lipoprotein cholesterol; ApoA_1_, apolipoprotein A_1_; ApoB, apolipoprotein B; Hcy, homocysteine; HHcy, hyperhomocysteinemia. *P < 0.05, significant differencea Serum levels of glutamine in tertiles: T1, <3.38; T2, 3.38-3.80; T3, >=3.80; | | | | |

| **Table S3. Differentially regulated genes in HBMECs treated with glutamine** | | | | | | |  |  |  |  |
| --- | --- | --- | --- | --- | --- | --- | --- | --- | --- | --- |
| Gene name | Treated01 | Treated02 | Treated03 | Ctrl01 | Ctrl02 | Ctrl03 | | log2FoldChange | pvalue | padj |
| A2M | 6495.317518 | 4620.798391 | 6389.506194 | 12558.0978 | 10671.47675 | 12498.77858 | | -1.029124365 | 9.58E-12 | 1.05E-07 |
| NEAT1 | 6206.616754 | 4657.385989 | 5489.933655 | 3158.751228 | 2691.751641 | 2731.956094 | | 0.930214095 | 1.31E-11 | 1.05E-07 |
| MAGED4 | 107.9255196 | 152.8070264 | 102.6630808 | 12.04569257 | 33.83347719 | 6.355849772 | | 2.794461927 | 2.83E-10 | 1.19E-06 |
| PHGDH | 473.0735274 | 567.1077671 | 443.1792397 | 239.0606679 | 254.7461812 | 253.1746826 | | 0.989325867 | 3.67E-10 | 1.19E-06 |
| CD34 | 3907.803187 | 3588.812909 | 3848.340829 | 5755.987865 | 5604.415986 | 6041.235208 | | -0.617008465 | 3.70E-10 | 1.19E-06 |
| UACA | 4015.728707 | 3999.885333 | 4054.683457 | 5410.369147 | 5883.044621 | 5835.729399 | | -0.504924614 | 1.15E-07 | 0.000306925 |
| APLNR | 116.0199335 | 115.1433227 | 100.6301485 | 226.0883836 | 211.9567835 | 202.3278844 | | -0.948787879 | 5.35E-07 | 0.001224926 |
| STAG3L1 | 88.1391743 | 116.2194286 | 44.72451043 | 23.1647934 | 24.87755675 | 21.18616591 | | 1.845362648 | 3.53E-06 | 0.006842244 |
| GJA5 | 60.25841508 | 66.71856083 | 78.26789325 | 134.3558017 | 143.2947269 | 148.3031613 | | -1.054893706 | 3.84E-06 | 0.006842244 |
| PLVAP | 159.1901413 | 111.9150053 | 107.7454115 | 264.0786448 | 244.7951585 | 229.8699001 | | -0.959766812 | 4.27E-06 | 0.006851048 |
| ACE | 5924.211644 | 3993.428698 | 5859.927332 | 9245.532343 | 7665.272787 | 9264.710351 | | -0.73020753 | 9.27E-06 | 0.013519533 |
| FABP4 | 1434.510031 | 2340.530158 | 1381.377493 | 2827.031387 | 3567.441639 | 2994.664551 | | -0.865008022 | 1.19E-05 | 0.015909842 |
| COL1A1 | 8.094413966 | 0 | 2.032932292 | 25.01797687 | 35.82368173 | 27.54201568 | | -3.065415783 | 2.71E-05 | 0.031937193 |
| GPIHBP1 | 95.33420894 | 66.71856083 | 89.44902086 | 163.0801456 | 166.1820791 | 152.5403945 | | -0.934083922 | 2.79E-05 | 0.031937193 |
| HS6ST1 | 1111.632851 | 896.396148 | 1159.787873 | 1640.993965 | 1454.839519 | 1566.716969 | | -0.557195553 | 4.08E-05 | 0.043664022 |
| AK5 | 676.3332559 | 787.7094602 | 644.4395366 | 459.5895011 | 515.4629759 | 448.0874089 | | 0.566476283 | 6.70E-05 | 0.063322248 |
| CLEC4GP1 | 244.6311777 | 229.2105396 | 172.7992448 | 140.8419439 | 106.4759429 | 90.0412051 | | 0.936332307 | 6.71E-05 | 0.063322248 |
| IL33 | 1296.005614 | 1077.181926 | 1149.623211 | 1693.809694 | 1577.237098 | 1608.029992 | | -0.469311672 | 8.47E-05 | 0.073297377 |
| MSS51 | 37.77393184 | 47.34865608 | 50.82330731 | 12.97228431 | 16.91673859 | 14.83031613 | | 1.603628736 | 8.68E-05 | 0.073297377 |
| MIR222HG | 186.1715212 | 217.3733756 | 179.9145079 | 113.0441918 | 114.4367611 | 119.7018374 | | 0.748310744 | 0.000109301 | 0.087642786 |
| ATG16L2 | 777.9631201 | 632.7502221 | 727.7897606 | 495.7265788 | 517.4531805 | 474.5701163 | | 0.524166662 | 0.000130611 | 0.099743438 |
| ITGB4 | 1194.37575 | 938.364275 | 1326.488321 | 776.4838748 | 684.6303619 | 820.9639288 | | 0.600589205 | 0.000137079 | 0.099924305 |
| UNC5B | 196.0646939 | 119.447746 | 181.9474402 | 315.967782 | 242.8049539 | 344.275196 | | -0.857092081 | 0.000153332 | 0.106912717 |
| GOLGA8B | 348.0598006 | 331.4405925 | 443.1792397 | 210.3363241 | 215.9371926 | 270.1236153 | | 0.69025791 | 0.000190746 | 0.12527282 |
| DLL4 | 1777.173555 | 1608.778201 | 1846.918987 | 2259.957244 | 2264.852767 | 2425.815996 | | -0.409212981 | 0.000195857 | 0.12527282 |
| FAM124B | 2466.997501 | 2103.786878 | 2551.330027 | 3236.584934 | 3163.430117 | 3165.213186 | | -0.425275773 | 0.000203099 | 0.12527282 |
| LAPTM5 | 1222.256509 | 1255.815492 | 1274.648547 | 1576.132543 | 1618.036291 | 1746.799379 | | -0.396747999 | 0.000218054 | 0.129515841 |
| SEMA5B | 24.2832419 | 3.22831746 | 16.26345834 | 75.98052236 | 50.75021578 | 39.19440693 | | -1.906987541 | 0.000360193 | 0.206300796 |
| GPNMB | 409.217595 | 412.148529 | 389.306534 | 598.5782615 | 505.5119532 | 606.9836532 | | -0.499018443 | 0.000378084 | 0.209080682 |
| CLDN11 | 2277.228463 | 2553.599111 | 2154.90823 | 1724.387221 | 1778.247757 | 1806.120643 | | 0.395939666 | 0.000442694 | 0.236649285 |
| AHSA2P | 904.7756056 | 899.6244654 | 824.3540445 | 679.1917426 | 667.7136233 | 611.2208864 | | 0.424561348 | 0.000497774 | 0.257509973 |
| COL13A1 | 1119.727265 | 964.1908146 | 957.5111096 | 809.8411773 | 609.9976916 | 713.973791 | | 0.511385667 | 0.000516667 | 0.258930821 |
| GSN | 23834.45161 | 23088.92647 | 23249.63016 | 29227.48313 | 29214.21245 | 28001.75548 | | -0.300837846 | 0.00060737 | 0.295163563 |
| TRBC2 | 520.7406318 | 611.2281057 | 461.4756303 | 366.9303275 | 388.0898854 | 348.5124291 | | 0.529188765 | 0.000658308 | 0.310508511 |
| DNAH8 | 13.49068994 | 8.608846559 | 9.148195315 | 0 | 0 | 1.059308295 | | 4.87678005 | 0.000698126 | 0.319881372 |
| MMP15 | 1326.584511 | 1178.335873 | 1361.04817 | 1809.633661 | 1640.923644 | 1623.919617 | | -0.392324518 | 0.000843254 | 0.375646404 |
| SHC2 | 297.6945581 | 303.4618412 | 273.4293933 | 443.8374416 | 395.0556013 | 389.8254527 | | -0.491154194 | 0.000867024 | 0.375796192 |
| AC007998.3 | 518.0424939 | 503.6175237 | 489.9366824 | 393.8014878 | 373.1633513 | 329.4448798 | | 0.462467796 | 0.000975207 | 0.395040482 |
| MAGED4B | 3.597517318 | 4.30442328 | 5.082330731 | 6.486142153 | 6.965715891 | 95.33774658 | | -3.065671289 | 0.00098902 | 0.395040482 |
| EHD3 | 2412.135362 | 1483.949926 | 2397.843639 | 3564.598409 | 2696.727152 | 3492.53945 | | -0.631722453 | 0.000993241 | 0.395040482 |
| UBAP1L | 70.15158771 | 48.4247619 | 42.69157814 | 23.1647934 | 14.92653405 | 27.54201568 | | 1.302801534 | 0.001027365 | 0.395040482 |
| FAM157A | 0.89937933 | 2.15221164 | 3.049398438 | 25.01797687 | 10.94612497 | 10.59308295 | | -2.958709464 | 0.001034589 | 0.395040482 |
| ESM1 | 2216.070668 | 2604.176084 | 2060.376878 | 3071.651605 | 3355.484855 | 2827.29384 | | -0.427872381 | 0.001076384 | 0.401441119 |
| CSGALNACT1 | 1916.577351 | 1728.225947 | 1922.137482 | 2362.808927 | 2354.411971 | 2317.76655 | | -0.337434383 | 0.001160302 | 0.422903745 |
| ABCA1 | 954.2414687 | 680.0988782 | 1104.898701 | 1365.796219 | 1207.059054 | 1533.878412 | | -0.583651737 | 0.001337642 | 0.476705924 |
| PALD1 | 2622.590125 | 2200.636402 | 2461.881006 | 3455.260584 | 2971.375379 | 3084.705756 | | -0.384530955 | 0.001373437 | 0.478821981 |
| SERPINE1 | 16431.66035 | 16360.03678 | 17064.43366 | 13029.73299 | 13709.52398 | 14024.18252 | | 0.29052174 | 0.001506933 | 0.514184865 |
| SORBS2 | 558.5145637 | 472.4104549 | 554.9905158 | 732.9340632 | 670.6989301 | 721.3889491 | | -0.421325557 | 0.001567803 | 0.523809475 |
| GOLGA8A | 554.9170464 | 505.7697354 | 514.3318699 | 414.186506 | 359.2319195 | 393.0033776 | | 0.433551993 | 0.001601653 | 0.524198195 |
| WSB1 | 13236.16559 | 11222.7076 | 11545.02249 | 10085.95105 | 9177.828238 | 8891.833831 | | 0.354752339 | 0.001778907 | 0.54738225 |
| GBP4 | 407.4188363 | 316.3751111 | 470.6238256 | 509.6254548 | 643.8311688 | 605.9243449 | | -0.55758685 | 0.001782203 | 0.54738225 |
| LINC01089 | 230.2411084 | 243.1999153 | 209.3920261 | 165.8599208 | 158.221261 | 134.5321535 | | 0.572340765 | 0.0018089 | 0.54738225 |
| AADAC | 73.74910503 | 75.32740739 | 64.0373672 | 42.62321986 | 35.82368173 | 32.83855715 | | 0.934275971 | 0.001837265 | 0.54738225 |
| PSAT1 | 489.2623553 | 616.6086348 | 525.5129975 | 395.6546713 | 392.0702944 | 403.5964605 | | 0.452459098 | 0.001843153 | 0.54738225 |
| NID2 | 781.5606374 | 413.2246348 | 730.8391591 | 1047.048662 | 880.6655091 | 1153.586734 | | -0.677110981 | 0.001899299 | 0.54838647 |
| NRARP | 1039.682505 | 1325.76237 | 996.1368232 | 1524.243406 | 1536.437905 | 1469.260606 | | -0.430999878 | 0.001914924 | 0.54838647 |
| AC097478.1 | 31.47827654 | 32.2831746 | 43.70804428 | 9.265917361 | 12.93632951 | 18.00824102 | | 1.426242715 | 0.001970743 | 0.554470165 |
| FOXP4 | 546.8226324 | 514.3785819 | 655.6206642 | 738.4936137 | 739.3609867 | 888.7596597 | | -0.462575166 | 0.002133604 | 0.579018261 |
| EFNB2 | 2089.258183 | 1545.287957 | 2062.40981 | 2724.179704 | 2366.353198 | 2663.101054 | | -0.444322331 | 0.002145836 | 0.579018261 |
| NTN4 | 5929.60792 | 5677.534306 | 5837.565077 | 7082.867231 | 7220.462072 | 6871.732912 | | -0.279547685 | 0.002166309 | 0.579018261 |
| MTHFD2 | 707.8115324 | 858.7324443 | 723.723896 | 463.295868 | 633.8801461 | 542.3658472 | | 0.482140504 | 0.002444069 | 0.632196193 |
| AC104083.1 | 12.59131061 | 18.29379894 | 11.18112761 | 30.57752729 | 44.77960216 | 32.83855715 | | -1.368464519 | 0.002444108 | 0.632196193 |
| MAFF | 837.3221559 | 774.7961903 | 726.7732945 | 666.2194582 | 525.4139987 | 528.5948393 | | 0.442731422 | 0.002540242 | 0.646632739 |
| SLC45A4 | 128.6112441 | 111.9150053 | 129.0912006 | 176.0524299 | 173.147795 | 208.6837342 | | -0.591407339 | 0.002637843 | 0.656598419 |
| CBSL | 0.89937933 | 2.15221164 | 9.148195315 | 63.93482979 | 93.5396134 | 7.415158067 | | -3.763364415 | 0.002661277 | 0.656598419 |
| IL1RL1 | 1162.897473 | 1206.314624 | 1213.660578 | 912.69286 | 949.3275657 | 992.5718727 | | 0.327979071 | 0.002767106 | 0.672364811 |
| ARL15 | 293.1976615 | 273.3308783 | 266.3141303 | 360.4441853 | 369.1829422 | 399.3592273 | | -0.437516768 | 0.002911396 | 0.696866479 |
| TMEM273 | 442.4946302 | 468.1060317 | 408.6193907 | 621.7430549 | 535.3650214 | 593.2126454 | | -0.408386798 | 0.003006215 | 0.705425309 |
| COL4A1 | 13049.09469 | 9717.235554 | 13088.0181 | 17561.69317 | 14655.86624 | 15944.70846 | | -0.425709985 | 0.003035128 | 0.705425309 |
| RNF144B | 1903.086661 | 1818.618836 | 2153.891764 | 2328.525033 | 2476.80955 | 2741.489868 | | -0.360919444 | 0.003131106 | 0.706025994 |
| SYNM | 768.0699475 | 703.7732062 | 757.2672788 | 977.5542816 | 913.503884 | 935.3692247 | | -0.342207933 | 0.003165531 | 0.706025994 |
| CPVL | 98.93172626 | 130.2088042 | 114.8606745 | 201.0704067 | 195.040045 | 144.0659282 | | -0.65539454 | 0.003169787 | 0.706025994 |
| ABCG2 | 734.7929123 | 843.6669628 | 788.7777294 | 934.00447 | 1090.632088 | 1036.003513 | | -0.370984686 | 0.003348187 | 0.735546208 |
| CHTF18 | 395.726905 | 356.1910264 | 363.8948803 | 297.4359473 | 258.7265902 | 265.8863821 | | 0.440803333 | 0.003480986 | 0.754386149 |
| LBH | 184.3727626 | 142.0459682 | 161.6181172 | 252.0329522 | 228.8735221 | 226.6919752 | | -0.533838859 | 0.0035377 | 0.756454679 |
| MRC1 | 15.2894486 | 16.1415873 | 24.39518751 | 37.99026118 | 47.76490897 | 40.25371522 | | -1.176785242 | 0.003622817 | 0.764462007 |
| AC009533.1 | 101.6298642 | 52.72918518 | 64.0373672 | 33.3573025 | 16.91673859 | 46.60956499 | | 1.179305763 | 0.003928465 | 0.818192171 |
| DIRC2 | 259.9206263 | 252.8848677 | 266.3141303 | 360.4441853 | 353.2613059 | 330.5041881 | | -0.422824822 | 0.004073335 | 0.837488177 |
| TGFBI | 245.530557 | 266.8742433 | 249.0342058 | 180.6853885 | 151.2555451 | 201.2685761 | | 0.514253687 | 0.004266888 | 0.858652829 |
| SPOCD1 | 618.7729788 | 550.9661798 | 501.11781 | 448.4704003 | 421.9233626 | 367.5799785 | | 0.432443037 | 0.004283359 | 0.858652829 |
| NFKBIZ | 154.6932447 | 115.1433227 | 126.0418021 | 96.36554055 | 65.67674983 | 81.56673874 | | 0.701298213 | 0.00459769 | 0.910285842 |
| MT2A | 3557.944628 | 5543.021078 | 3321.811365 | 2316.47934 | 3320.656276 | 2741.489868 | | 0.568117462 | 0.004710731 | 0.913233611 |
| PTPRG | 966.8327793 | 821.0687406 | 1053.058927 | 1259.238169 | 1125.460668 | 1356.973926 | | -0.396802904 | 0.004726469 | 0.913233611 |
| INSIG1 | 1258.231682 | 1467.808338 | 1315.307193 | 1025.737052 | 1133.421486 | 1036.003513 | | 0.338528063 | 0.004810982 | 0.918496574 |
| TM4SF18 | 4110.163536 | 4325.945396 | 4156.330071 | 4849.781147 | 5082.982396 | 5209.678196 | | -0.266045229 | 0.005126983 | 0.967310981 |
| NALT1 | 45.86834581 | 31.20706878 | 41.67511199 | 20.38501819 | 19.9020454 | 11.65239125 | | 1.190145543 | 0.005280008 | 0.984598758 |
| ELMOD1 | 66.55407039 | 75.32740739 | 76.23496096 | 37.06366944 | 49.75511351 | 36.01648204 | | 0.826835473 | 0.005373517 | 0.989127534 |
| CPPED1 | 1310.395683 | 1425.840211 | 1417.970274 | 1715.121303 | 1720.531825 | 1650.402324 | | -0.292413138 | 0.00544564 | 0.989127534 |
| MEG8 | 48.5664838 | 59.1858201 | 58.95503647 | 29.65093555 | 34.82857946 | 19.06754932 | | 0.990533005 | 0.005489328 | 0.989127534 |
| SELPLG | 23.38386257 | 12.91326984 | 29.47751824 | 52.81572896 | 31.84327265 | 68.85503919 | | -1.216719276 | 0.005552394 | 0.989374845 |
| PCDH12 | 1719.613278 | 1377.415449 | 1822.5238 | 2069.079347 | 2043.940063 | 2264.801135 | | -0.37409865 | 0.005819928 | 0.999784719 |
| SEMA6B | 12173.09923 | 14259.47822 | 12562.5051 | 15702.95015 | 16987.39085 | 15351.49582 | | -0.301060716 | 0.005820508 | 0.999784719 |
| AC126755.2 | 57.5602771 | 86.08846559 | 27.44458594 | 21.31160993 | 31.84327265 | 18.00824102 | | 1.262825074 | 0.005897152 | 0.999784719 |
| ZNF692 | 360.6511112 | 372.3326137 | 300.8739792 | 265.0052365 | 254.7461812 | 241.5222913 | | 0.441004225 | 0.006053994 | 0.999784719 |
| LIFR | 1827.538798 | 1450.590645 | 1973.977256 | 2272.002937 | 2196.19071 | 2340.012024 | | -0.374038757 | 0.006349222 | 0.999784719 |
| CHERP | 552.2189084 | 448.7361269 | 632.2419429 | 727.3745128 | 683.6352596 | 784.9474468 | | -0.42637093 | 0.006653202 | 0.999784719 |
| HYPK | 55.76151844 | 37.6637037 | 57.93857033 | 25.94456861 | 16.91673859 | 30.71994056 | | 1.045230436 | 0.006910498 | 0.999784719 |
| NOSTRIN | 55.76151844 | 68.87077247 | 64.0373672 | 85.24643972 | 131.3534997 | 96.39705487 | | -0.732160493 | 0.006966211 | 0.999784719 |
| ABI3BP | 735.6922916 | 427.2140105 | 722.7074299 | 460.5160928 | 329.3788514 | 457.6211836 | | 0.596794326 | 0.007180444 | 0.999784719 |
| LRCH1 | 483.8660793 | 403.5396825 | 532.6282606 | 627.3026053 | 590.0956462 | 654.6525265 | | -0.397844832 | 0.007317202 | 0.999784719 |
| ACCS | 328.2734553 | 275.4830899 | 259.1988673 | 225.1617919 | 199.020454 | 201.2685761 | | 0.465115385 | 0.007353279 | 0.999784719 |
| TIMP3 | 1404.830513 | 1457.04728 | 1514.534558 | 1720.680854 | 1743.419177 | 1859.086058 | | -0.282541532 | 0.007388262 | 0.999784719 |
| SIRPB2 | 45.86834581 | 31.20706878 | 44.72451043 | 76.9071141 | 59.70613621 | 80.50743044 | | -0.829111937 | 0.007388886 | 0.999784719 |
| ACER2 | 305.7889721 | 184.0140952 | 376.0924741 | 445.6906251 | 423.9135671 | 452.3246421 | | -0.609106031 | 0.007458393 | 0.999784719 |
| HCP5 | 40.47206983 | 50.57697354 | 40.65864584 | 83.39325625 | 67.66695437 | 73.09227237 | | -0.771326589 | 0.007482216 | 0.999784719 |
| RRAS | 1063.965747 | 1349.436698 | 1030.696672 | 870.9962319 | 952.3128726 | 793.4219132 | | 0.395657817 | 0.007622885 | 0.999784719 |
| FRMD5 | 380.4374564 | 344.3538624 | 414.7181876 | 290.0232134 | 289.5747606 | 284.9539314 | | 0.398727337 | 0.007784018 | 0.999784719 |
| FAM184B | 5.396275978 | 8.608846559 | 5.082330731 | 0 | 0 | 1.059308295 | | 4.158330953 | 0.007953382 | 1 |
| NSUN5P1 | 174.4795899 | 190.4707301 | 173.815711 | 137.1355769 | 126.3779883 | 114.4052959 | | 0.509208464 | 0.00801421 | 0.999784719 |
| SCN4A | 2.698137989 | 2.15221164 | 0 | 11.11910083 | 11.94122724 | 8.474466362 | | -2.683719563 | 0.008037094 | 0.999784719 |
| CDK10 | 1248.338509 | 1510.852571 | 1250.25336 | 1031.296602 | 1146.357815 | 933.2506081 | | 0.365456361 | 0.008075308 | 0.999784719 |
| ARTN | 6.295655307 | 5.3805291 | 7.115263023 | 0 | 0.99510227 | 0 | | 4.140053376 | 0.008118999 | 1 |
| TRIM24 | 1277.118648 | 1334.371217 | 1320.389524 | 1546.481608 | 1574.251791 | 1620.741692 | | -0.270187165 | 0.008452982 | 0.999784719 |
| EMG1 | 366.0473872 | 387.3980952 | 335.4338282 | 261.2988696 | 296.5404765 | 266.9456904 | | 0.400678674 | 0.008617183 | 0.999784719 |
| SEMA3G | 236.5367637 | 152.8070264 | 233.7872136 | 352.1048597 | 274.6482266 | 287.072548 | | -0.550848057 | 0.008708466 | 0.999784719 |
| MAMDC4 | 74.64848436 | 89.31678305 | 59.97150262 | 46.3295868 | 45.77470443 | 37.07579034 | | 0.790266996 | 0.008780055 | 0.999784719 |
| APOE | 260.8200056 | 348.6582857 | 209.3920261 | 423.4524234 | 404.0115217 | 350.6310457 | | -0.526548116 | 0.00897608 | 0.999784719 |
| IFIT3 | 144.8000721 | 116.2194286 | 151.4534558 | 177.9056133 | 189.0694313 | 234.1071333 | | -0.54008869 | 0.008988324 | 0.999784719 |
| TBXA2R | 53.06338045 | 77.47961903 | 59.97150262 | 97.29213229 | 93.5396134 | 114.4052959 | | -0.683624292 | 0.009075174 | 0.999784719 |
| TSPAN11 | 777.0637408 | 529.4440634 | 747.1026174 | 1018.324318 | 780.1601798 | 1059.308295 | | -0.475835617 | 0.009075226 | 0.999784719 |
| LIMCH1 | 1819.444384 | 1401.089778 | 1901.808159 | 2272.929529 | 2014.086995 | 2377.087815 | | -0.379228492 | 0.009126876 | 0.999784719 |
| DRAXIN | 25.18262123 | 15.06548148 | 31.51045053 | 46.3295868 | 46.7698067 | 50.84679817 | | -0.998910338 | 0.009220522 | 0.999784719 |
| HDAC10 | 51.26462179 | 66.71856083 | 43.70804428 | 24.09138514 | 30.84817038 | 30.71994056 | | 0.917108288 | 0.009253374 | 0.999784719 |
| AC011481.1 | 7.195034637 | 8.608846559 | 3.049398438 | 0 | 0.99510227 | 0 | | 4.143607576 | 0.009269913 | 1 |
| RCAN2 | 120.5168302 | 117.2955344 | 112.8277422 | 185.3183472 | 150.2604428 | 166.3114024 | | -0.517724012 | 0.009287222 | 0.999784719 |
| PLEKHA2 | 65.65469106 | 67.79466665 | 56.92210418 | 90.80599014 | 108.4661474 | 98.51567146 | | -0.644796661 | 0.009389423 | 0.999784719 |
| RAB30 | 321.9778 | 298.0813121 | 323.2362345 | 430.8651573 | 387.0947831 | 401.4778439 | | -0.370243436 | 0.009413601 | 0.999784719 |
| TCF7L1 | 558.5145637 | 526.2157459 | 608.8632215 | 657.8801326 | 707.5177141 | 812.4894625 | | -0.362011845 | 0.009828412 | 0.999784719 |
| DDIT4L | 38.67331117 | 83.93625395 | 46.75744272 | 121.3835174 | 85.57879523 | 95.33774658 | | -0.843940097 | 0.009930412 | 0.999784719 |
| PGGHG | 670.0376006 | 532.6723809 | 580.4021694 | 491.0936201 | 387.0947831 | 469.2735748 | | 0.404913835 | 0.010025339 | 0.999784719 |
| AL136038.4 | 2.698137989 | 1.07610582 | 3.049398438 | 13.89887604 | 18.90694313 | 4.237233181 | | -2.431425693 | 0.010049146 | 0.999784719 |
| RDM1P3 | 3.597517318 | 4.30442328 | 5.082330731 | 0 | 0 | 0 | | 4.566146101 | 0.010104738 | 1 |
| HMBS | 244.6311777 | 331.4405925 | 250.0506719 | 207.5565489 | 205.9861699 | 172.6672521 | | 0.492162113 | 0.010128102 | 0.999784719 |
| ALDH5A1 | 66.55407039 | 44.12033862 | 58.95503647 | 99.14531576 | 82.59348842 | 94.27843828 | | -0.697713408 | 0.010352408 | 0.999784719 |
| RASGRP3 | 1086.45023 | 1049.203174 | 1054.075394 | 1234.220192 | 1271.740701 | 1373.922859 | | -0.282103183 | 0.01055459 | 0.999784719 |
| MIR29B2CHG | 166.385176 | 94.69731215 | 153.4863881 | 94.51235708 | 84.58369296 | 81.56673874 | | 0.671472798 | 0.010557459 | 0.999784719 |
| N4BP3 | 851.7122251 | 617.6847406 | 828.4199091 | 1130.441918 | 918.4793954 | 999.9870307 | | -0.407420457 | 0.010610755 | 0.999784719 |
| AC093690.1 | 9.893172626 | 7.532740739 | 9.148195315 | 0.926591736 | 0.99510227 | 2.118616591 | | 2.73814359 | 0.010613573 | 0.999784719 |
| FAM238C | 14.39006927 | 8.608846559 | 19.31285678 | 2.779775208 | 3.980409081 | 4.237233181 | | 1.953239464 | 0.01105661 | 0.999784719 |
| RLF | 256.3231089 | 238.895492 | 235.8201459 | 340.9857589 | 282.6090447 | 351.690354 | | -0.415002534 | 0.011085742 | 0.999784719 |
| PDK4 | 542.3257358 | 546.6617565 | 568.2045757 | 656.9535409 | 701.5471005 | 691.7283168 | | -0.306936062 | 0.011090268 | 0.999784719 |
| PPM1H | 35.07579385 | 41.96812698 | 44.72451043 | 69.49438021 | 83.58859069 | 56.14333965 | | -0.785155052 | 0.011174482 | 0.999784719 |
| ELN | 20.68572458 | 17.21769312 | 27.44458594 | 49.10936201 | 41.79429535 | 38.13509863 | | -0.982450306 | 0.011270856 | 0.999784719 |
| VEPH1 | 1045.97816 | 1086.866878 | 1108.964565 | 865.4366815 | 903.5528613 | 904.6492842 | | 0.277962639 | 0.011283 | 0.999784719 |
| DAPK2 | 71.95034637 | 57.03360846 | 65.05383335 | 105.6314579 | 84.58369296 | 115.4646042 | | -0.65209246 | 0.011351581 | 0.999784719 |
| HIST1H4I | 73.74910503 | 87.16457141 | 53.87270574 | 43.5498116 | 42.78939762 | 38.13509863 | | 0.784779448 | 0.011564677 | 0.999784719 |
| STRIP2 | 542.3257358 | 386.3219893 | 541.7764559 | 366.0037358 | 355.2515105 | 372.8765199 | | 0.427724786 | 0.011673959 | 0.999784719 |
| MASP1 | 34.17641453 | 50.57697354 | 50.82330731 | 78.76029757 | 73.63756799 | 73.09227237 | | -0.7403244 | 0.011727311 | 0.999784719 |
| SNCA-AS1 | 8.094413966 | 11.83716402 | 3.049398438 | 0.926591736 | 0.99510227 | 0 | | 3.545918206 | 0.011730379 | 1 |
| VAV3 | 71.05096704 | 90.39288887 | 81.31729169 | 126.0164761 | 125.382886 | 110.1680627 | | -0.578862426 | 0.011741628 | 0.999784719 |
| FAM13B | 292.2982821 | 275.4830899 | 301.8904454 | 378.9760201 | 357.241715 | 380.291678 | | -0.360021212 | 0.012370703 | 0.999784719 |
| CD99P1 | 420.9095263 | 485.3237248 | 411.6687892 | 343.7655341 | 346.29559 | 336.8600379 | | 0.358959243 | 0.012405736 | 0.999784719 |
| UBR3 | 586.3953229 | 495.0086772 | 623.0937476 | 738.4936137 | 684.6303619 | 743.6344233 | | -0.345486073 | 0.01246009 | 0.999784719 |
| AC008147.4 | 3.597517318 | 6.456634919 | 7.115263023 | 0.926591736 | 0 | 0 | | 4.004407912 | 0.012665703 | 1 |
| DPH6 | 99.83110559 | 91.46899469 | 90.465487 | 63.00823805 | 61.69634075 | 59.32126454 | | 0.615045073 | 0.012674442 | 0.999784719 |
| ACYP1 | 75.54786369 | 87.16457141 | 89.44902086 | 62.08164632 | 50.75021578 | 42.37233181 | | 0.694687311 | 0.012693642 | 0.999784719 |
| COL4A2 | 12660.56282 | 9153.356104 | 12634.6742 | 16190.3374 | 13551.30272 | 14777.35072 | | -0.369925776 | 0.012746758 | 0.999784719 |
| ROBO4 | 6444.952276 | 6703.063152 | 7230.123697 | 7616.584071 | 8165.809229 | 8704.336262 | | -0.264959084 | 0.01275783 | 0.999784719 |
| FGF2 | 646.653738 | 639.206857 | 541.7764559 | 775.5572831 | 749.3120094 | 760.583356 | | -0.322290122 | 0.012834781 | 0.999784719 |
| RNF180 | 32.37765587 | 30.13096296 | 32.52691668 | 50.03595375 | 55.72572713 | 60.38057283 | | -0.803295326 | 0.012957324 | 0.999784719 |
| FZD1 | 186.1715212 | 129.1326984 | 171.7827787 | 251.1063605 | 212.9518858 | 223.5140503 | | -0.495008442 | 0.012962697 | 0.999784719 |
| SNHG1 | 535.1307011 | 667.1856083 | 476.7226225 | 421.5992399 | 415.9527489 | 436.4350177 | | 0.397641061 | 0.01301615 | 0.999784719 |
| MECOM | 1541.536171 | 1472.112762 | 1673.103276 | 1816.119803 | 1907.611052 | 1925.822481 | | -0.269364117 | 0.013214826 | 0.999784719 |
| HCRTR1 | 37.77393184 | 36.58759788 | 47.77390887 | 19.45842646 | 18.90694313 | 24.36409079 | | 0.963839386 | 0.013311794 | 0.999784719 |
| MGARP | 214.9516598 | 258.2653968 | 183.9803724 | 164.933329 | 147.275136 | 156.7776277 | | 0.485349612 | 0.013396146 | 0.999784719 |
| KAT7 | 2176.497978 | 1996.176296 | 2448.666946 | 2591.677086 | 2662.893675 | 2876.022022 | | -0.29603322 | 0.013518988 | 0.999784719 |
| AC092171.3 | 14.39006927 | 19.36990476 | 12.19759375 | 32.43071076 | 29.85306811 | 34.95717374 | | -1.085172679 | 0.013772237 | 0.999784719 |
| PTP4A3 | 354.3554559 | 358.343238 | 299.8575131 | 408.6269556 | 515.4629759 | 413.1302352 | | -0.401005537 | 0.013859866 | 0.999784719 |
| SPSB3 | 27.88075922 | 34.43538624 | 12.19759375 | 10.1925091 | 9.951022702 | 7.415158067 | | 1.430545217 | 0.013904143 | 0.999784719 |
| GJA4 | 110.6236575 | 156.0353439 | 172.7992448 | 203.8501819 | 230.8637267 | 197.0313429 | | -0.526918651 | 0.0140604 | 0.999784719 |
| AL359538.1 | 3.597517318 | 4.30442328 | 4.065864584 | 0 | 0 | 0 | | 4.448807036 | 0.014170096 | 1 |
| COX18 | 286.0026268 | 299.1574179 | 291.7257839 | 216.8224662 | 239.8196471 | 216.0988922 | | 0.381958364 | 0.014281205 | 0.999784719 |
| HEXIM2 | 44.96896648 | 38.73980952 | 52.8562396 | 29.65093555 | 18.90694313 | 23.3047825 | | 0.923826465 | 0.01447283 | 0.999784719 |
| MEG3 | 5948.494886 | 3588.812909 | 5552.954556 | 4096.462065 | 2998.24314 | 3820.925021 | | 0.467334883 | 0.014511991 | 0.999784719 |
| RUSC1-AS1 | 116.0199335 | 96.84952379 | 125.025336 | 63.93482979 | 66.6718521 | 90.0412051 | | 0.619110132 | 0.014512335 | 0.999784719 |
| MICALL2 | 590.8922196 | 570.3360846 | 594.6326955 | 487.3872532 | 450.7813284 | 480.9259661 | | 0.307309512 | 0.014535632 | 0.999784719 |
| ADAMTS4 | 959.6377447 | 796.3183067 | 1021.548477 | 751.465898 | 645.8213733 | 774.3543639 | | 0.355513479 | 0.014615509 | 0.999784719 |
| SLC16A14 | 71.95034637 | 49.50086772 | 51.83977345 | 77.83370583 | 102.4955338 | 98.51567146 | | -0.679502235 | 0.01467485 | 0.999784719 |
| CXCR4 | 318.3802827 | 361.5715555 | 310.0221746 | 421.5992399 | 487.6001124 | 383.4696029 | | -0.386013854 | 0.014711031 | 0.999784719 |
| PEX5L | 8.094413966 | 6.456634919 | 6.098796877 | 0 | 0.99510227 | 1.059308295 | | 3.369225825 | 0.014789901 | 1 |
| MEX3D | 1729.506451 | 2003.709037 | 1760.519365 | 2103.363241 | 2293.710733 | 2256.326669 | | -0.276536649 | 0.014795094 | 0.999784719 |
| AC079447.1 | 8.094413966 | 5.3805291 | 7.115263023 | 0.926591736 | 0 | 1.059308295 | | 3.380223364 | 0.014876032 | 1 |
| MEG9 | 111.5230369 | 111.9150053 | 103.6795469 | 68.56778847 | 80.60328388 | 72.03296408 | | 0.565122792 | 0.014993801 | 0.999784719 |
| SIRPA | 2064.075561 | 1845.521481 | 2206.748003 | 2483.265853 | 2373.318914 | 2555.051608 | | -0.276906394 | 0.015112682 | 0.999784719 |
| AC020916.1 | 285.1032475 | 225.9822222 | 311.0386407 | 197.3640398 | 201.0106586 | 208.6837342 | | 0.439033634 | 0.015272719 | 0.999784719 |
| AC015813.1 | 222.1466944 | 252.8848677 | 256.1494688 | 136.2089852 | 198.0253518 | 189.6161849 | | 0.482117686 | 0.015342482 | 0.999784719 |
| SNRPCP19 | 1.798758659 | 4.30442328 | 6.098796877 | 0 | 0 | 0 | | 4.472849963 | 0.015445928 | 1 |
| CFAP45 | 17.08820726 | 13.98937566 | 13.2140599 | 5.559550417 | 3.980409081 | 4.237233181 | | 1.68247656 | 0.015501379 | 0.999784719 |
| PTTG1 | 596.2884955 | 871.6457141 | 531.6117944 | 465.1490515 | 536.3601236 | 437.494326 | | 0.473665615 | 0.015540484 | 0.999784719 |
| MAPRE3 | 112.4224162 | 81.78404231 | 92.4984193 | 136.2089852 | 147.275136 | 133.4728452 | | -0.536463725 | 0.015562173 | 0.999784719 |
| NPIPA1 | 89.03855363 | 118.3716402 | 126.0418021 | 58.37527937 | 80.60328388 | 76.27019726 | | 0.631133429 | 0.015571936 | 0.999784719 |
| AC079174.2 | 8.094413966 | 10.7610582 | 7.115263023 | 2.779775208 | 0 | 1.059308295 | | 2.728288353 | 0.015607192 | 1 |
| AC106745.1 | 0 | 0 | 0 | 2.779775208 | 5.970613621 | 3.177924886 | | -4.441274015 | 0.015623799 | 1 |
| ZNF362 | 697.0189804 | 704.849312 | 701.3616408 | 815.4007278 | 864.7438728 | 866.5141855 | | -0.275824072 | 0.015738153 | 0.999784719 |
| HERC5 | 101.6298642 | 130.2088042 | 109.7783438 | 158.4471869 | 187.0792268 | 143.0066199 | | -0.519202826 | 0.015771113 | 0.999784719 |
| BANK1 | 8.094413966 | 8.608846559 | 8.131729169 | 25.01797687 | 16.91673859 | 21.18616591 | | -1.348387939 | 0.015827901 | 0.999784719 |
| FILIP1 | 751.8811196 | 471.3343491 | 681.0323179 | 915.4726353 | 739.3609867 | 951.2588492 | | -0.451519477 | 0.016030622 | 0.999784719 |
| IFIH1 | 236.5367637 | 305.6140529 | 264.281198 | 344.6921258 | 325.3984423 | 400.4185356 | | -0.409873649 | 0.016077654 | 0.999784719 |
| SUMO2P19 | 0 | 0 | 0 | 4.63295868 | 1.99020454 | 5.296541476 | | -4.440120184 | 0.016158286 | 1 |
| GALC | 449.6896648 | 476.7148782 | 465.5414949 | 574.4868764 | 569.1984985 | 568.8485546 | | -0.299559879 | 0.016180868 | 0.999784719 |
| SLC9A3-AS1 | 151.0957274 | 157.1114497 | 119.9430052 | 122.3101092 | 83.58859069 | 72.03296408 | | 0.619695126 | 0.016351793 | 0.999784719 |
| NSFP1 | 7.195034637 | 62.41413755 | 47.77390887 | 19.45842646 | 7.960818161 | 10.59308295 | | 1.616195531 | 0.016422597 | 0.999784719 |
| CAPN10-DT | 55.76151844 | 38.73980952 | 50.82330731 | 35.21048597 | 20.89714767 | 20.12685761 | | 0.927308214 | 0.016530032 | 0.999784719 |
| GPX3 | 1092.745885 | 1202.010201 | 979.8733648 | 872.8494154 | 935.396134 | 835.794245 | | 0.308250569 | 0.016684695 | 0.999784719 |
| HAGHL | 136.7056581 | 176.4813545 | 101.6466146 | 103.7782744 | 80.60328388 | 83.68535533 | | 0.627060995 | 0.016716168 | 0.999784719 |
| NOMO3 | 400.2238017 | 250.732656 | 447.2451043 | 549.4688995 | 605.0221803 | 429.0198596 | | -0.5272764 | 0.016782938 | 0.999784719 |
| VEGFA | 376.8399391 | 316.3751111 | 323.2362345 | 294.6561721 | 241.8098517 | 227.7512835 | | 0.411134303 | 0.016799403 | 0.999784719 |
| HMGCS1 | 741.0885676 | 758.654603 | 727.7897606 | 632.8621557 | 614.973203 | 579.4416375 | | 0.285215547 | 0.016842432 | 0.999784719 |
| KIAA1551 | 1220.45775 | 992.169566 | 1356.982305 | 1646.553515 | 1359.309701 | 1560.361119 | | -0.354991462 | 0.017130563 | 0.999784719 |
| PDGFD | 310.2858687 | 386.3219893 | 324.2527006 | 414.186506 | 435.8547943 | 460.7991084 | | -0.361763908 | 0.017235066 | 0.999784719 |
| MN1 | 266.2162816 | 249.6565502 | 326.2856329 | 388.2419374 | 352.2662036 | 361.2241287 | | -0.387927479 | 0.017592919 | 0.999784719 |
| RBM6 | 1420.119961 | 1165.422603 | 1247.203961 | 1055.387987 | 1038.88677 | 1043.418671 | | 0.289289043 | 0.017747638 | 0.999784719 |
| SULT1B1 | 578.3009089 | 412.148529 | 599.7150262 | 406.7737721 | 376.1486581 | 415.2488518 | | 0.409430104 | 0.017868618 | 0.999784719 |
| MYL9 | 576.5021503 | 644.5873861 | 578.3692371 | 712.5490451 | 775.1846685 | 720.3296408 | | -0.295798577 | 0.018175605 | 0.999784719 |
| RPL29P11 | 58.45965642 | 60.26192592 | 65.05383335 | 80.61348104 | 56.7208294 | 238.3443664 | | -1.029427964 | 0.018470694 | 0.999784719 |
| SAMHD1 | 1014.499884 | 1066.420868 | 1165.88667 | 1219.394725 | 1403.094201 | 1332.609835 | | -0.284760106 | 0.018607544 | 0.999784719 |
| CAPG | 1318.490097 | 1511.928677 | 1090.668175 | 1087.818698 | 1041.872077 | 954.4367741 | | 0.345939609 | 0.018766817 | 0.999784719 |
| AP001972.5 | 1225.854026 | 1408.622518 | 1561.292 | 1529.802956 | 1965.326984 | 1811.417185 | | -0.338982134 | 0.018790334 | 0.999784719 |
| NPIPA7 | 4.496896648 | 20.44601058 | 11.18112761 | 0.926591736 | 0.99510227 | 5.296541476 | | 2.342203829 | 0.018791441 | 0.999784719 |
| RARRES1 | 33.2770352 | 47.34865608 | 20.32932292 | 18.53183472 | 8.955920432 | 16.94893272 | | 1.180309247 | 0.018919044 | 0.999784719 |
| COL6A1 | 1456.994514 | 1111.617312 | 1327.504787 | 1729.02018 | 1478.721973 | 1675.825723 | | -0.325342913 | 0.019019445 | 0.999784719 |
| LINC02099 | 0.89937933 | 3.22831746 | 7.115263023 | 19.45842646 | 11.94122724 | 10.59308295 | | -1.92363377 | 0.019413356 | 0.999784719 |
| RAC3 | 867.9010531 | 1135.29164 | 847.7327659 | 690.3108434 | 837.8761115 | 663.1269928 | | 0.378976015 | 0.019470575 | 0.999784719 |
| ADAMTSL1 | 557.6151844 | 361.5715555 | 475.7061564 | 335.4262085 | 308.4817038 | 385.5882195 | | 0.440225612 | 0.019485079 | 0.999784719 |
| SMO | 354.3554559 | 307.7662645 | 339.4996928 | 449.396992 | 382.1192717 | 445.9687923 | | -0.349993682 | 0.019517155 | 0.999784719 |
| CATSPER2 | 43.17020782 | 22.59822222 | 28.46105209 | 13.89887604 | 9.951022702 | 19.06754932 | | 1.144638399 | 0.019543056 | 0.999784719 |
| PDE4D | 392.1293877 | 372.3326137 | 482.8214194 | 490.1670284 | 506.5070555 | 650.4152933 | | -0.400360221 | 0.019963936 | 0.999784719 |
| CENPK | 179.8758659 | 235.6671746 | 156.5357865 | 132.5026183 | 134.3388065 | 138.7693867 | | 0.494795746 | 0.020076547 | 0.999784719 |
| CCDC14 | 894.882433 | 737.1324866 | 807.07412 | 694.0172103 | 650.7968847 | 629.2291274 | | 0.305642796 | 0.020085255 | 0.999784719 |
| GCOM1 | 420.0101469 | 380.9414602 | 484.8543517 | 353.9580432 | 341.3200787 | 299.7842476 | | 0.369234782 | 0.020087881 | 0.999784719 |
| MAPK8IP3 | 3026.411444 | 1978.958603 | 2738.359798 | 2243.278593 | 1738.443666 | 1890.865307 | | 0.399191295 | 0.020107793 | 0.999784719 |
| MYO15B | 794.151948 | 462.7255026 | 641.3901382 | 546.6891243 | 367.1927377 | 427.9605513 | | 0.50094499 | 0.020192437 | 0.999784719 |
| HSD3B7 | 110.6236575 | 143.122074 | 130.1076667 | 83.39325625 | 98.51512475 | 83.68535533 | | 0.52898711 | 0.02027849 | 0.999784719 |
| SRA1 | 479.3691827 | 479.9431957 | 469.6073595 | 397.5078548 | 380.1290672 | 380.291678 | | 0.303106226 | 0.0203239 | 0.999784719 |
| ALDH3B1 | 46.76772514 | 40.89202116 | 39.6421797 | 23.1647934 | 28.85796584 | 15.88962443 | | 0.904546232 | 0.020375323 | 0.999784719 |
| KLF2 | 1216.860233 | 1883.185185 | 1380.361026 | 927.5183278 | 1303.583974 | 1088.968928 | | 0.432108079 | 0.020439626 | 0.999784719 |
| MVD | 896.6811916 | 1165.422603 | 823.3375783 | 778.3370583 | 772.1993617 | 686.4317753 | | 0.366384821 | 0.020441071 | 0.999784719 |
| AL590648.3 | 0.89937933 | 0 | 0 | 3.706366944 | 4.975511351 | 6.355849772 | | -3.811091741 | 0.020496744 | 1 |
| OLFML2A | 21.58510391 | 22.59822222 | 23.37872136 | 39.84344465 | 33.83347719 | 51.90610647 | | -0.893104888 | 0.020499218 | 0.999784719 |
| HERC6 | 194.2659352 | 206.6123174 | 201.2602969 | 267.7850117 | 252.7559766 | 257.4119158 | | -0.370570505 | 0.020522294 | 0.999784719 |
| MYH10 | 6553.777175 | 4704.734645 | 7020.731671 | 7912.166834 | 7228.422891 | 8348.408675 | | -0.361666055 | 0.020597619 | 0.999784719 |
| SMAD3 | 2753.000128 | 2237.224 | 2819.677089 | 2144.133277 | 2029.013529 | 2207.598487 | | 0.291860415 | 0.020652559 | 0.999784719 |
| AC110749.1 | 1.798758659 | 5.3805291 | 4.065864584 | 0 | 0 | 0 | | 4.353580823 | 0.020701033 | 1 |
| UCHL3 | 29.67951788 | 51.65307936 | 37.60924741 | 20.38501819 | 22.88735221 | 18.00824102 | | 0.950209819 | 0.020907594 | 0.999784719 |
| WHAMM | 312.0846274 | 299.1574179 | 327.302099 | 421.5992399 | 372.168249 | 388.7661444 | | -0.333630004 | 0.020937241 | 0.999784719 |
| NSUN6 | 120.5168302 | 96.84952379 | 104.696013 | 76.9071141 | 74.63267026 | 68.85503919 | | 0.548499592 | 0.021065993 | 0.999784719 |
| MIDN | 1146.708645 | 1029.83327 | 1259.401555 | 1321.319816 | 1402.099099 | 1469.260606 | | -0.286718584 | 0.021066126 | 0.999784719 |
| ADGRF5 | 4002.238017 | 3051.836105 | 4109.572629 | 4762.681523 | 4261.027921 | 4972.393138 | | -0.326021705 | 0.021102379 | 0.999784719 |
| POSTN | 195.1653145 | 233.5149629 | 176.8651094 | 148.2546778 | 142.2996246 | 154.6590111 | | 0.442845784 | 0.021262357 | 0.999784719 |
| AL009031.1 | 8.993793296 | 1.07610582 | 6.098796877 | 0 | 0 | 1.059308295 | | 3.931730107 | 0.0212737 | 1 |
| LRRC37A5P | 4.496896648 | 9.684952379 | 9.148195315 | 2.779775208 | 0 | 0 | | 3.004688869 | 0.021374762 | 1 |
| AC017048.3 | 7.195034637 | 5.3805291 | 15.24699219 | 1.853183472 | 2.985306811 | 0 | | 2.50427857 | 0.021391424 | 0.999784719 |
| SLCO4A1 | 64.75531173 | 68.87077247 | 81.31729169 | 41.69662812 | 41.79429535 | 51.90610647 | | 0.668095272 | 0.021503133 | 0.999784719 |
| KAZALD1 | 29.67951788 | 34.43538624 | 31.51045053 | 12.97228431 | 15.92163632 | 19.06754932 | | 0.99894221 | 0.021592037 | 0.999784719 |
| ALDOC | 319.279662 | 316.3751111 | 291.7257839 | 237.2074844 | 253.7510789 | 236.2257498 | | 0.351066818 | 0.021659412 | 0.999784719 |
| AL355987.5 | 9.893172626 | 9.684952379 | 10.16466146 | 3.706366944 | 0.99510227 | 2.118616591 | | 2.111627075 | 0.021705215 | 0.999784719 |
| SH3BP5-AS1 | 169.083314 | 143.122074 | 202.2767631 | 119.530334 | 101.5004316 | 139.828695 | | 0.513475348 | 0.021883455 | 0.999784719 |
| SV2A | 204.1591078 | 163.5680846 | 212.4414245 | 238.1340762 | 282.6090447 | 254.2339909 | | -0.415733535 | 0.021943626 | 0.999784719 |
| AC116407.4 | 1.798758659 | 7.532740739 | 6.098796877 | 0 | 0 | 1.059308295 | | 3.846226524 | 0.022038383 | 1 |
| LINC01235 | 476.6710447 | 463.8016084 | 406.5864584 | 380.8292035 | 335.349465 | 352.7496623 | | 0.33356226 | 0.022066242 | 0.999784719 |
| AJUBA | 759.9755335 | 677.9466665 | 744.053219 | 902.5003509 | 855.7879523 | 874.9886519 | | -0.270740805 | 0.022240169 | 0.999784719 |
| EML4 | 608.8798061 | 468.1060317 | 597.6820939 | 670.8524169 | 741.3511913 | 702.3213998 | | -0.335212639 | 0.022385737 | 0.999784719 |
| PLAUR | 482.9667 | 612.3042115 | 491.9696147 | 424.3790151 | 435.8547943 | 384.5289112 | | 0.349155394 | 0.022445285 | 0.999784719 |
| ARSG | 277.0088335 | 274.4069841 | 303.9233777 | 214.042691 | 221.9078062 | 230.9292084 | | 0.35954829 | 0.022497 | 0.999784719 |
| STC2 | 189.7690385 | 125.9043809 | 196.1779662 | 139.9153521 | 83.58859069 | 115.4646042 | | 0.594998565 | 0.022611865 | 0.999784719 |
| MYO18A | 1176.388163 | 719.9147935 | 1187.232459 | 1492.739287 | 1282.686826 | 1370.744934 | | -0.426636851 | 0.022624456 | 0.999784719 |
| AC009041.4 | 3.597517318 | 6.456634919 | 8.131729169 | 17.60524299 | 12.93632951 | 20.12685761 | | -1.48903249 | 0.022675066 | 0.999784719 |
| LRRC4 | 58.45965642 | 44.12033862 | 78.26789325 | 97.29213229 | 95.52981794 | 88.9818968 | | -0.638875408 | 0.02269894 | 0.999784719 |
| SNHG12 | 175.3789693 | 218.4494814 | 166.700448 | 132.5026183 | 149.2653405 | 124.9983788 | | 0.460642824 | 0.022748569 | 0.999784719 |
| FTX | 442.4946302 | 446.5839153 | 491.9696147 | 318.7475572 | 338.3347719 | 419.4860849 | | 0.360505452 | 0.02287287 | 0.999784719 |
| MTSS1 | 1182.683818 | 897.4722538 | 1141.491482 | 1435.290599 | 1199.098236 | 1434.303432 | | -0.336161782 | 0.022911945 | 0.999784719 |
| AL451085.2 | 7.195034637 | 3.22831746 | 2.032932292 | 15.75205951 | 8.955920432 | 18.00824102 | | -1.754589318 | 0.023079025 | 0.999784719 |
| SLC30A3 | 298.5939374 | 340.0494391 | 318.1539037 | 416.0396895 | 358.2368173 | 445.9687923 | | -0.351549305 | 0.02316862 | 0.999784719 |
| FAM171B | 565.7095983 | 543.4334391 | 642.4066043 | 713.4756368 | 689.6058732 | 744.6937316 | | -0.294028168 | 0.02354297 | 0.999784719 |
| PROS1 | 884.089881 | 794.1660951 | 747.1026174 | 989.5999741 | 942.3618499 | 1010.580114 | | -0.278152311 | 0.023732884 | 0.999784719 |
| SNRPA1 | 304.8895927 | 423.985693 | 291.7257839 | 232.5745258 | 233.8490335 | 284.9539314 | | 0.441342218 | 0.023737126 | 0.999784719 |
| TEX30 | 202.3603492 | 234.5910687 | 172.7992448 | 146.4014943 | 155.2359541 | 148.3031613 | | 0.437730077 | 0.023760211 | 0.999784719 |
| FER1L4 | 114.2211749 | 71.02298411 | 102.6630808 | 50.03595375 | 39.80409081 | 82.62604703 | | 0.745572611 | 0.023810454 | 0.999784719 |
| ENPP2 | 58.45965642 | 57.03360846 | 71.15263023 | 95.43894882 | 86.57389751 | 95.33774658 | | -0.571827726 | 0.023866504 | 0.999784719 |
| LENG8 | 3677.562079 | 2158.668275 | 3349.255951 | 2522.182706 | 1951.395552 | 2343.189949 | | 0.430470603 | 0.023884231 | 0.999784719 |
| AP000695.2 | 320.1790413 | 397.0830475 | 364.9113465 | 281.6838878 | 273.6531243 | 292.3690895 | | 0.351020872 | 0.024056186 | 0.999784719 |
| KITLG | 222.1466944 | 207.6884232 | 218.5402214 | 259.4456861 | 280.6188402 | 292.3690895 | | -0.359070855 | 0.024117499 | 0.999784719 |
| AL133297.2 | 0 | 0 | 1.016466146 | 8.339325625 | 0.99510227 | 6.355849772 | | -3.877645859 | 0.024180055 | 1 |
| RASGRF2 | 40.47206983 | 34.43538624 | 59.97150262 | 55.59550417 | 76.6228748 | 96.39705487 | | -0.758136385 | 0.024432565 | 0.999784719 |
| SGSM3 | 25.18262123 | 25.82653968 | 14.23052605 | 6.486142153 | 10.94612497 | 9.533774658 | | 1.280189731 | 0.02461596 | 0.999784719 |
| AC022400.7 | 160.0895207 | 116.2194286 | 126.0418021 | 97.29213229 | 99.51022702 | 84.74466362 | | 0.516971277 | 0.024733951 | 0.999784719 |
| EMP3 | 939.8513994 | 1486.102137 | 937.1817867 | 742.1999806 | 912.5087817 | 842.1500948 | | 0.42924408 | 0.024946578 | 0.999784719 |
| ST20 | 34.17641453 | 43.0442328 | 46.75744272 | 12.04569257 | 26.86776129 | 25.42339909 | | 0.950888328 | 0.024963953 | 0.999784719 |
| CHST1 | 132.2087615 | 114.0672169 | 89.44902086 | 156.5940034 | 171.1575905 | 146.1845447 | | -0.4953741 | 0.025033764 | 0.999784719 |
| OAS3 | 276.1094542 | 181.8618836 | 309.0057084 | 405.8471804 | 294.550272 | 378.1730614 | | -0.490670914 | 0.025198083 | 0.999784719 |
| PPP1R3B | 1746.594658 | 1407.546412 | 1706.646659 | 1943.989462 | 1951.395552 | 1983.025129 | | -0.273741684 | 0.025212724 | 0.999784719 |
| COL12A1 | 52.16400112 | 34.43538624 | 55.90563804 | 91.73258187 | 57.71593167 | 84.74466362 | | -0.713674133 | 0.025239092 | 0.999784719 |
| TMEM100 | 1.798758659 | 1.07610582 | 1.016466146 | 12.97228431 | 5.970613621 | 4.237233181 | | -2.565510098 | 0.025471412 | 1 |
| ROBO3 | 329.1728346 | 276.5591957 | 291.7257839 | 227.0149753 | 255.7412834 | 209.7430425 | | 0.374890262 | 0.025519968 | 0.999784719 |
| ENTPD7 | 249.1280743 | 210.9167407 | 277.4952579 | 338.2059837 | 276.6384311 | 362.283437 | | -0.404738458 | 0.025582912 | 0.999784719 |
| STRBP | 321.9778 | 278.7114074 | 338.4832267 | 411.4067308 | 409.9821353 | 367.5799785 | | -0.339902554 | 0.025609845 | 0.999784719 |
| AC131971.1 | 7.195034637 | 11.83716402 | 8.131729169 | 0.926591736 | 2.985306811 | 2.118616591 | | 2.178197744 | 0.025616012 | 0.999784719 |
| AC090425.2 | 0.89937933 | 7.532740739 | 7.115263023 | 0 | 0 | 1.059308295 | | 3.856507212 | 0.025696506 | 1 |
| AC093525.8 | 20.68572458 | 32.2831746 | 28.46105209 | 13.89887604 | 11.94122724 | 12.71169954 | | 1.072690562 | 0.025751051 | 0.999784719 |
| PDHA1 | 995.6129179 | 1076.10582 | 915.8359976 | 873.7760071 | 809.0181457 | 780.7102136 | | 0.277652857 | 0.025923953 | 0.999784719 |
| ACAP1 | 25.18262123 | 18.29379894 | 28.46105209 | 12.97228431 | 2.985306811 | 12.71169954 | | 1.32823247 | 0.02593832 | 0.999784719 |
| ZBTB46 | 295.8957994 | 277.6353015 | 312.0551069 | 409.5535474 | 331.369056 | 385.5882195 | | -0.347154806 | 0.026263307 | 0.999784719 |
| MSMO1 | 987.5185039 | 1112.693418 | 963.6099065 | 824.6666451 | 823.9446797 | 891.9375846 | | 0.270056864 | 0.026392203 | 0.999784719 |
| KIF22 | 681.7295318 | 783.4050369 | 700.3451747 | 618.036688 | 603.0319757 | 550.8403135 | | 0.288241869 | 0.026395376 | 0.999784719 |
| DPM2 | 589.9928402 | 734.980275 | 594.6326955 | 498.506354 | 561.2376804 | 456.5618753 | | 0.339145664 | 0.026530919 | 0.999784719 |
| EME2 | 218.5491771 | 178.6335661 | 249.0342058 | 180.6853885 | 129.3632951 | 156.7776277 | | 0.468971017 | 0.026535804 | 0.999784719 |
| SLC17A9 | 394.8275257 | 361.5715555 | 368.977211 | 313.1880068 | 309.476806 | 274.3608485 | | 0.327029097 | 0.026598752 | 0.999784719 |
| IFI27L1 | 199.6622112 | 242.1238095 | 201.2602969 | 173.2726546 | 156.2310564 | 154.6590111 | | 0.407010055 | 0.026713613 | 0.999784719 |
| HEMK1 | 302.1914547 | 343.2777566 | 292.7422501 | 252.959544 | 202.0057608 | 263.7677655 | | 0.383979072 | 0.026784693 | 0.999784719 |
| SNCAIP | 428.1045609 | 321.7556402 | 428.9487137 | 533.71684 | 456.751942 | 526.4762228 | | -0.362576506 | 0.026933667 | 0.999784719 |
| SOCS3 | 1238.445337 | 927.6032168 | 1136.409151 | 942.3437956 | 830.9103956 | 879.2258851 | | 0.316734483 | 0.027024674 | 0.999784719 |
| MST1 | 177.1777279 | 143.122074 | 144.3381927 | 125.0898844 | 114.4367611 | 92.15982169 | | 0.485914423 | 0.027060377 | 0.999784719 |
| DLC1 | 1166.494991 | 1037.36601 | 1396.624485 | 1473.28086 | 1447.873803 | 1519.048095 | | -0.302245691 | 0.027134934 | 0.999784719 |
| GLUD1P3 | 55.76151844 | 46.27255026 | 57.93857033 | 27.79775208 | 24.87755675 | 41.31302352 | | 0.773248216 | 0.027250156 | 0.999784719 |
| CASKIN2 | 761.7742922 | 646.7395978 | 761.3331434 | 903.4269427 | 820.9593729 | 923.7168335 | | -0.286631359 | 0.027738844 | 0.999784719 |
| REXO5 | 167.2845553 | 173.253037 | 169.7498464 | 132.5026183 | 130.3583974 | 119.7018374 | | 0.414325812 | 0.027751156 | 0.999784719 |
| ELMO1 | 728.497257 | 667.1856083 | 796.9094585 | 891.3812501 | 806.0328388 | 1029.647663 | | -0.314142545 | 0.027924202 | 0.999784719 |
| PRR26 | 2.698137989 | 1.07610582 | 7.115263023 | 0 | 0 | 0 | | 4.314657119 | 0.02796701 | 1 |
| MEGF9 | 511.7468385 | 450.8883385 | 589.5503647 | 661.5864996 | 582.1348281 | 722.4482574 | | -0.340500823 | 0.028028692 | 0.999784719 |
| OBSCN | 111.5230369 | 95.77341797 | 114.8606745 | 168.639696 | 113.4416588 | 183.2603351 | | -0.52897796 | 0.028128582 | 0.999784719 |
| SLC25A13 | 455.9853201 | 456.2688676 | 601.7479585 | 605.9909954 | 651.791987 | 655.7118348 | | -0.337925575 | 0.028187117 | 0.999784719 |
| LRP3 | 443.3940095 | 502.5414179 | 516.3648022 | 561.5145921 | 643.8311688 | 594.2719537 | | -0.300122152 | 0.028513875 | 0.999784719 |
| SORT1 | 1018.99678 | 793.0899893 | 1150.639677 | 1294.448655 | 1166.259861 | 1283.881654 | | -0.337462903 | 0.028528856 | 0.999784719 |
| PODXL | 5820.783021 | 4549.775407 | 6161.817778 | 6792.844017 | 6327.855336 | 7249.905973 | | -0.30106413 | 0.028757693 | 0.999784719 |
| ZNF519 | 39.5726905 | 30.13096296 | 32.52691668 | 21.31160993 | 15.92163632 | 16.94893272 | | 0.916009717 | 0.028845365 | 0.999784719 |
| CYFIP2 | 743.7867056 | 601.5431533 | 916.8524638 | 981.2606485 | 893.6018386 | 1038.122129 | | -0.364363116 | 0.028885407 | 0.999784719 |
| SUN3 | 6.295655307 | 4.30442328 | 3.049398438 | 0 | 0.99510227 | 0 | | 3.682452134 | 0.028888656 | 1 |
| MT1E | 397.5256637 | 402.4635766 | 315.1045053 | 319.6741489 | 291.5649652 | 239.4036747 | | 0.389371546 | 0.028912291 | 0.999784719 |
| RAB3IL1 | 114.2211749 | 91.46899469 | 88.43255471 | 134.3558017 | 138.3192156 | 136.6507701 | | -0.473431501 | 0.028977982 | 0.999784719 |
| ORC6 | 194.2659352 | 227.058328 | 165.6839818 | 151.034453 | 144.2898292 | 139.828695 | | 0.43033831 | 0.028994317 | 0.999784719 |
| AL353804.2 | 1.798758659 | 4.30442328 | 4.065864584 | 0 | 0 | 0 | | 4.209563973 | 0.029044081 | 1 |
| ANKRD35 | 126.8124855 | 101.1539471 | 96.56428388 | 85.24643972 | 54.73062486 | 77.32950556 | | 0.579830559 | 0.029085392 | 0.999784719 |
| ACHE | 0.89937933 | 0 | 1.016466146 | 3.706366944 | 9.951022702 | 4.237233181 | | -3.188003235 | 0.029126908 | 1 |
| PNMA2 | 132.2087615 | 136.6654391 | 117.9100729 | 100.0719075 | 83.58859069 | 94.27843828 | | 0.476058003 | 0.029133885 | 0.999784719 |
| SNX18 | 536.0300804 | 521.9113227 | 612.9290861 | 717.1820037 | 619.9487143 | 721.3889491 | | -0.301045324 | 0.029242122 | 0.999784719 |
| ACP4 | 12.59131061 | 0 | 13.2140599 | 1.853183472 | 0.99510227 | 0 | | 3.153190615 | 0.029295004 | 1 |
| RAB27B | 17.98758659 | 33.35928042 | 21.34578907 | 11.11910083 | 11.94122724 | 9.533774658 | | 1.149389996 | 0.02938096 | 0.999784719 |
| NUAK1 | 2860.925647 | 2020.92673 | 2859.319269 | 3534.020881 | 2919.630061 | 3363.303838 | | -0.342487007 | 0.029489873 | 0.999784719 |
| NFYC-AS1 | 11.69193128 | 13.98937566 | 21.34578907 | 5.559550417 | 3.980409081 | 7.415158067 | | 1.47362005 | 0.029550258 | 0.999784719 |
| GCAT | 593.5903575 | 821.0687406 | 570.237508 | 459.5895011 | 508.4972601 | 558.2554716 | | 0.378649707 | 0.029773999 | 0.999784719 |
| CD38 | 9.893172626 | 3.22831746 | 11.18112761 | 19.45842646 | 15.92163632 | 26.48270738 | | -1.334150096 | 0.029784105 | 0.999784719 |
| SLC25A27 | 92.63607095 | 85.01235977 | 89.44902086 | 57.44868764 | 44.77960216 | 74.15158067 | | 0.601988359 | 0.029820885 | 0.999784719 |
| DLAT | 637.6599447 | 640.2829628 | 673.9170549 | 556.8816334 | 548.3013509 | 516.9424481 | | 0.266472884 | 0.029887332 | 0.999784719 |
| LINC02206 | 1.798758659 | 3.22831746 | 5.082330731 | 0 | 0 | 0 | | 4.202579477 | 0.030119736 | 1 |
| ETV4 | 259.0212469 | 307.7662645 | 282.5775886 | 189.0247142 | 203.9959654 | 254.2339909 | | 0.392769084 | 0.030168084 | 0.999784719 |
| SRF | 822.0327073 | 755.4262856 | 877.2102841 | 973.8479146 | 936.3912362 | 1041.300054 | | -0.265464375 | 0.030352028 | 0.999784719 |
| B3GNT5 | 384.9343531 | 313.1467936 | 364.9113465 | 454.0299507 | 422.9184648 | 449.1467172 | | -0.317655366 | 0.030712278 | 0.999784719 |
| EXOC3L1 | 177.1777279 | 166.7964021 | 144.3381927 | 210.3363241 | 218.9224994 | 209.7430425 | | -0.3868959 | 0.030778744 | 0.999784719 |
| PRICKLE2 | 182.5740039 | 154.9592381 | 207.3590938 | 216.8224662 | 247.7804653 | 256.3526075 | | -0.40208419 | 0.030783358 | 0.999784719 |
| SCUBE2 | 67.45344972 | 34.43538624 | 54.88917189 | 75.98052236 | 99.51022702 | 76.27019726 | | -0.67656088 | 0.030865504 | 0.999784719 |
| ZCCHC2 | 902.0774676 | 690.8599364 | 941.2476513 | 1157.313078 | 963.2589975 | 1066.723453 | | -0.330342596 | 0.03109929 | 0.999784719 |
| GATA3 | 474.872286 | 457.3449735 | 541.7764559 | 602.2846285 | 580.1446235 | 617.5767362 | | -0.288163465 | 0.031174556 | 0.999784719 |
| QPCTL | 410.1169743 | 495.0086772 | 402.5205939 | 328.0134746 | 354.2564082 | 360.1648204 | | 0.326508832 | 0.03132031 | 0.999784719 |
| HOXA-AS2 | 219.4485564 | 143.122074 | 228.7048829 | 159.3737786 | 117.4220679 | 139.828695 | | 0.506108383 | 0.031341523 | 0.999784719 |
| DNPH1 | 715.9059464 | 669.33782 | 546.8587866 | 579.1198351 | 498.5462374 | 427.9605513 | | 0.359270455 | 0.031365182 | 0.999784719 |
| STK10 | 1677.34245 | 1269.804867 | 1672.08681 | 1978.273357 | 1766.30653 | 1955.483113 | | -0.302886964 | 0.031558249 | 0.999784719 |
| MX1 | 107.0261402 | 115.1433227 | 118.9265391 | 142.6951274 | 160.2114655 | 155.7183194 | | -0.427578362 | 0.031609157 | 0.999784719 |
| PLEKHG1 | 1081.053954 | 772.6439787 | 1077.454115 | 1325.952774 | 1147.352918 | 1228.797623 | | -0.336307166 | 0.031693052 | 0.999784719 |
| ATAD3B | 391.2300084 | 412.148529 | 408.6193907 | 302.9954977 | 360.2270218 | 310.3773305 | | 0.315773729 | 0.031701934 | 0.999784719 |
| ZNF213-AS1 | 113.3217955 | 110.8388995 | 116.8936068 | 77.83370583 | 84.58369296 | 82.62604703 | | 0.477731959 | 0.031710772 | 0.999784719 |
| SPTBN5 | 789.6550514 | 543.4334391 | 666.8017918 | 581.8996103 | 503.5217487 | 463.9770333 | | 0.368672132 | 0.031781988 | 0.999784719 |
| EDNRB | 166.385176 | 142.0459682 | 187.0297709 | 214.9692828 | 213.9469881 | 222.454742 | | -0.393446199 | 0.032057397 | 0.999784719 |
| FDFT1 | 2960.756753 | 3769.598687 | 2987.394003 | 2565.732517 | 2890.772095 | 2496.789652 | | 0.288823333 | 0.032198518 | 0.999784719 |
| RHBDL2 | 25.18262123 | 20.44601058 | 28.46105209 | 8.339325625 | 11.94122724 | 14.83031613 | | 1.086150054 | 0.03253538 | 0.999784719 |
| GTF2IP4 | 587.2947022 | 524.0635343 | 531.6117944 | 732.0074715 | 724.4344527 | 580.5009458 | | -0.310156001 | 0.032766142 | 0.999784719 |
| GUCY1B1 | 73.74910503 | 82.86014813 | 106.7289453 | 129.7228431 | 119.4122724 | 122.8797623 | | -0.500933691 | 0.032827268 | 0.999784719 |
| ABLIM1 | 4146.13871 | 2631.07873 | 4400.281946 | 5084.208856 | 4344.616512 | 5224.508512 | | -0.39046078 | 0.032862959 | 0.999784719 |
| CLK2 | 657.4462899 | 589.7059893 | 598.6985601 | 538.3497987 | 507.5021578 | 474.5701163 | | 0.279857989 | 0.033177968 | 0.999784719 |
| CDCA3 | 220.3479358 | 269.026455 | 196.1779662 | 167.7131042 | 174.1428973 | 176.9044853 | | 0.401336014 | 0.033219936 | 0.999784719 |
| ATOH8 | 475.7716654 | 462.7255026 | 459.442698 | 599.5048532 | 498.5462374 | 628.1698191 | | -0.303943787 | 0.033224394 | 0.999784719 |
| CDH12 | 19.78634525 | 5.3805291 | 18.29639063 | 3.706366944 | 3.980409081 | 6.355849772 | | 1.644953493 | 0.033437772 | 0.999784719 |
| ITGB3BP | 181.6746246 | 205.5362116 | 197.1944323 | 138.9887604 | 162.20167 | 144.0659282 | | 0.391490728 | 0.033612623 | 0.999784719 |
| SLC9A1 | 1320.288856 | 1291.326984 | 1601.950646 | 1595.59097 | 1630.972621 | 1951.24588 | | -0.297086096 | 0.033784168 | 0.999784719 |
| ZP3 | 44.06958715 | 36.58759788 | 34.55984897 | 13.89887604 | 22.88735221 | 26.48270738 | | 0.873925316 | 0.034186446 | 0.999784719 |
| NEK2 | 239.2349017 | 281.9397248 | 235.8201459 | 201.0704067 | 197.0302495 | 191.7348014 | | 0.358373653 | 0.034413886 | 0.999784719 |
| DIAPH3 | 333.6697313 | 322.831746 | 347.631422 | 247.3999935 | 279.6237379 | 278.5980817 | | 0.318767869 | 0.034497421 | 0.999784719 |
| AC027348.1 | 2.698137989 | 7.532740739 | 3.049398438 | 0 | 0.99510227 | 0 | | 3.630738462 | 0.034615057 | 1 |
| BNIP3P27 | 2.698137989 | 6.456634919 | 1.016466146 | 0 | 0 | 0 | | 4.209984521 | 0.034842587 | 1 |
| INSR | 209.5553838 | 160.3397672 | 240.9024766 | 238.1340762 | 275.6433288 | 307.1994056 | | -0.424351319 | 0.034894862 | 0.999784719 |
| AC006333.2 | 58.45965642 | 69.94687829 | 64.0373672 | 47.25617854 | 41.79429535 | 33.89786545 | | 0.640926577 | 0.035118742 | 0.999784719 |
| AFG3L1P | 171.781452 | 162.4919788 | 248.0177397 | 162.1535538 | 118.4171702 | 133.4728452 | | 0.490462997 | 0.035192978 | 0.999784719 |
| MSL3P1 | 0.89937933 | 0 | 0 | 3.706366944 | 5.970613621 | 3.177924886 | | -3.588687155 | 0.0355777 | 1 |
| PARP11 | 83.64227765 | 94.69731215 | 104.696013 | 135.2823935 | 123.3926815 | 129.235612 | | -0.45709586 | 0.035586128 | 0.999784719 |
| FSTL5 | 5.396275978 | 9.684952379 | 18.29639063 | 4.63295868 | 2.985306811 | 1.059308295 | | 1.920260454 | 0.035666118 | 0.999784719 |
| AL359762.3 | 0.89937933 | 2.15221164 | 2.032932292 | 8.339325625 | 10.94612497 | 4.237233181 | | -2.233347962 | 0.036044484 | 1 |
| SULF2 | 3974.357258 | 2703.17782 | 3926.608722 | 4754.342198 | 3962.49724 | 4725.574305 | | -0.341965589 | 0.036059422 | 0.999784719 |
| UBE2C | 557.6151844 | 718.8386877 | 496.0354793 | 456.8097259 | 487.6001124 | 447.0281006 | | 0.348330585 | 0.036101827 | 0.999784719 |
| CYP2AC1P | 0 | 1.07610582 | 1.016466146 | 3.706366944 | 8.955920432 | 4.237233181 | | -3.063726932 | 0.036183679 | 1 |
| PSEN2 | 342.6635246 | 322.831746 | 317.1374376 | 415.1130978 | 375.1535559 | 418.4267766 | | -0.298071586 | 0.036263798 | 0.999784719 |
| FSIP2 | 12.59131061 | 4.30442328 | 17.27992448 | 5.559550417 | 0.99510227 | 2.118616591 | | 1.964818654 | 0.036280269 | 0.999784719 |
| CLEC11A | 468.5766307 | 711.305947 | 378.1254064 | 349.3250845 | 426.8988739 | 351.690354 | | 0.46519566 | 0.036666637 | 0.999784719 |
| PSMG4 | 351.6573179 | 431.5184338 | 311.0386407 | 312.2614151 | 260.7167948 | 273.3015402 | | 0.369094236 | 0.036749268 | 0.999784719 |
| GTF2IP20 | 110.6236575 | 90.39288887 | 140.2723282 | 64.86142153 | 87.56899978 | 81.56673874 | | 0.547031832 | 0.036911469 | 0.999784719 |
| CCDC18-AS1 | 112.4224162 | 92.54510051 | 98.59721617 | 94.51235708 | 44.77960216 | 54.02472306 | | 0.647946724 | 0.036974604 | 0.999784719 |
| AC005332.3 | 35.97517318 | 24.75043386 | 32.52691668 | 12.04569257 | 17.91184086 | 19.06754932 | | 0.936580671 | 0.03700459 | 0.999784719 |
| GPR89B | 73.74910503 | 75.32740739 | 47.77390887 | 107.4846414 | 236.8343403 | 41.31302352 | | -0.97064294 | 0.03739227 | 0.999784719 |
| HECW2 | 2590.212469 | 1825.075471 | 2881.681524 | 3172.650104 | 2796.237379 | 3352.710755 | | -0.353039177 | 0.037396134 | 0.999784719 |
| CFAP54 | 502.7530453 | 417.5290581 | 517.3812684 | 426.2321986 | 336.3445673 | 379.2323697 | | 0.332568269 | 0.037515986 | 0.999784719 |
| HES4 | 191.5677972 | 347.5821798 | 199.2273646 | 357.6644101 | 358.2368173 | 313.5552554 | | -0.482276754 | 0.037554697 | 0.999784719 |
| MRC2 | 1584.706379 | 1231.065058 | 1535.880347 | 1884.687591 | 1676.747325 | 1720.316672 | | -0.279082837 | 0.037682456 | 0.999784719 |
| CCDC78 | 17.98758659 | 13.98937566 | 12.19759375 | 4.63295868 | 6.965715891 | 5.296541476 | | 1.392148892 | 0.037697322 | 0.999784719 |
| AL645940.1 | 0 | 0 | 0 | 4.63295868 | 3.980409081 | 1.059308295 | | -4.144839195 | 0.037818454 | 1 |
| TMEM63B | 840.0202939 | 703.7732062 | 859.9303596 | 897.8673923 | 952.3128726 | 1094.265469 | | -0.29172629 | 0.037818594 | 0.999784719 |
| THSD7A | 580.9990469 | 361.5715555 | 603.7808908 | 706.0629029 | 621.9389189 | 719.2703325 | | -0.403699231 | 0.037837645 | 0.999784719 |
| KCNK6 | 956.9396067 | 784.4811427 | 853.8315627 | 1161.019445 | 1006.048395 | 985.1567146 | | -0.280132386 | 0.037863486 | 0.999784719 |
| SPDL1 | 337.2672486 | 369.1042962 | 358.8125496 | 305.7752729 | 285.5943515 | 268.0049987 | | 0.308118965 | 0.03806983 | 0.999784719 |
| AC010186.3 | 17.98758659 | 15.06548148 | 19.31285678 | 7.412733889 | 4.975511351 | 9.533774658 | | 1.26152435 | 0.038109204 | 0.999784719 |
| SGTB | 200.5615905 | 158.1875555 | 206.3426277 | 233.5011175 | 239.8196471 | 260.5898406 | | -0.374694364 | 0.038179096 | 0.999784719 |
| PIR | 1948.955007 | 2604.176084 | 1780.848688 | 1685.470368 | 1842.929404 | 1483.031613 | | 0.337499251 | 0.038480616 | 0.999784719 |
| LINC02352 | 4.496896648 | 5.3805291 | 1.016466146 | 12.04569257 | 14.92653405 | 8.474466362 | | -1.70376172 | 0.038846733 | 0.999784719 |
| RF00003 | 0.89937933 | 0 | 1.016466146 | 8.339325625 | 1.99020454 | 6.355849772 | | -3.091184916 | 0.03902302 | 1 |
| GUCY1A1 | 64.75531173 | 59.1858201 | 77.2514271 | 105.6314579 | 87.56899978 | 95.33774658 | | -0.520635633 | 0.03903038 | 0.999784719 |
| PLAC8 | 330.072214 | 441.2033862 | 339.4996928 | 292.8029886 | 314.4523174 | 253.1746826 | | 0.366386906 | 0.039051313 | 0.999784719 |
| FUS | 3709.939735 | 3362.830687 | 2797.314834 | 2926.176703 | 2745.487163 | 2414.163605 | | 0.287650208 | 0.03947499 | 0.999784719 |
| AC010973.2 | 1.798758659 | 8.608846559 | 12.19759375 | 0 | 0 | 3.177924886 | | 2.87510437 | 0.039672019 | 1 |
| P4HA3 | 954.2414687 | 781.2528253 | 826.3869768 | 757.0254484 | 705.5275096 | 645.1187518 | | 0.281816543 | 0.039699844 | 0.999784719 |
| FAM117A | 520.7406318 | 555.2706031 | 588.5338986 | 634.7153392 | 648.8066802 | 721.3889491 | | -0.268414603 | 0.039856813 | 0.999784719 |
| HMCN2 | 0.89937933 | 0 | 1.016466146 | 9.265917361 | 6.965715891 | 1.059308295 | | -3.149015942 | 0.03999358 | 1 |
| ELFN1 | 49.46586313 | 38.73980952 | 41.67511199 | 69.49438021 | 66.6718521 | 61.43988113 | | -0.60256491 | 0.040088037 | 0.999784719 |
| EIF3CL | 108.8248989 | 269.026455 | 738.9708882 | 89.8793984 | 159.2163632 | 127.1169954 | | 1.569754478 | 0.040368232 | 0.999784719 |
| PCK2 | 405.6200777 | 532.6723809 | 417.767586 | 337.2793919 | 381.1241695 | 359.1055121 | | 0.330713703 | 0.040639103 | 0.999784719 |
| AL390728.6 | 189.7690385 | 264.7220317 | 165.6839818 | 148.2546778 | 168.1722837 | 133.4728452 | | 0.460828375 | 0.040716315 | 0.999784719 |
| SYNPO | 6838.880422 | 4740.246137 | 7519.816549 | 8175.318887 | 7274.197595 | 8850.520807 | | -0.347363909 | 0.040852116 | 0.999784719 |
| ANKRD36B | 90.83731229 | 52.72918518 | 70.13616408 | 51.88913722 | 33.83347719 | 48.72818158 | | 0.672163774 | 0.041041263 | 0.999784719 |
| KIAA0895 | 52.16400112 | 68.87077247 | 48.79037501 | 88.02621493 | 70.65226118 | 93.21912999 | | -0.57155353 | 0.041106895 | 0.999784719 |
| CCNA2 | 786.9569134 | 915.7660527 | 836.5516382 | 638.4217062 | 787.1258957 | 661.0083763 | | 0.282873029 | 0.041394803 | 0.999784719 |
| E4F1 | 645.7543587 | 630.5980105 | 550.9246512 | 493.8733953 | 518.4482828 | 504.2307486 | | 0.269327143 | 0.041556043 | 0.999784719 |
| FUT8 | 670.0376006 | 701.6209946 | 699.3287085 | 495.7265788 | 592.0858508 | 619.6953527 | | 0.279096405 | 0.041558184 | 0.999784719 |
| LCMT2 | 81.84351899 | 75.32740739 | 75.21849481 | 119.530334 | 91.54940886 | 114.4052959 | | -0.48524803 | 0.042103096 | 0.999784719 |
| GIPC3 | 134.9068994 | 162.4919788 | 145.3546589 | 154.7408199 | 211.9567835 | 231.9885167 | | -0.434903893 | 0.042124421 | 0.999784719 |
| GATD3A | 460.4822168 | 530.5201692 | 626.143146 | 426.2321986 | 452.7715329 | 418.4267766 | | 0.316885136 | 0.042139192 | 0.999784719 |
| ADRA1B | 8.094413966 | 5.3805291 | 11.18112761 | 17.60524299 | 21.89224994 | 16.94893272 | | -1.191456645 | 0.042306746 | 0.999784719 |
| CYP2U1 | 294.0970408 | 314.2228994 | 314.0880391 | 350.2516762 | 377.1437604 | 406.7743854 | | -0.297968745 | 0.042358578 | 0.999784719 |
| LINC01762 | 1.798758659 | 5.3805291 | 2.032932292 | 0 | 0 | 0 | | 4.065474343 | 0.04295921 | 1 |
| TMEM42 | 377.7393184 | 429.3662221 | 299.8575131 | 290.9498051 | 301.5159879 | 273.3015402 | | 0.35385188 | 0.042986827 | 0.999784719 |
| PLPP5 | 583.6971849 | 577.8688253 | 604.7973569 | 531.8636565 | 475.6588851 | 462.917725 | | 0.263794533 | 0.043031885 | 0.999784719 |
| NOTCH2NLA | 8.993793296 | 3.22831746 | 12.19759375 | 1.853183472 | 0 | 3.177924886 | | 2.295112191 | 0.043241734 | 1 |
| AL033397.2 | 0 | 0 | 0 | 6.486142153 | 1.99020454 | 1.059308295 | | -4.126847095 | 0.043360554 | 1 |
| MXD3 | 402.0225603 | 467.0299258 | 337.4667605 | 320.6007407 | 343.3102832 | 282.8353148 | | 0.348722974 | 0.043370416 | 0.999784719 |
| AL109613.1 | 0.89937933 | 1.07610582 | 2.032932292 | 4.63295868 | 5.970613621 | 9.533774658 | | -2.331905638 | 0.043376805 | 1 |
| NR3C1 | 420.0101469 | 331.4405925 | 398.4547293 | 449.396992 | 454.7617375 | 527.5355311 | | -0.314215676 | 0.043586241 | 0.999784719 |
| ARHGAP45 | 411.915733 | 469.1821375 | 465.5414949 | 589.3123442 | 599.0515666 | 473.510808 | | -0.30497494 | 0.043607214 | 0.999784719 |
| AC007325.2 | 2.698137989 | 1.07610582 | 0 | 2.779775208 | 17.91184086 | 3.177924886 | | -2.625247024 | 0.043609041 | 1 |
| AL591806.3 | 5.396275978 | 3.22831746 | 11.18112761 | 19.45842646 | 16.91673859 | 13.77100784 | | -1.341165057 | 0.043662083 | 0.999784719 |
| AL157938.2 | 3.597517318 | 3.22831746 | 5.082330731 | 0 | 0 | 1.059308295 | | 3.481100343 | 0.043838856 | 1 |
| MTFR2 | 67.45344972 | 100.0778413 | 67.08676564 | 39.84344465 | 63.68654529 | 47.66887329 | | 0.632312073 | 0.043860664 | 0.999784719 |
| SPART-AS1 | 9.893172626 | 10.7610582 | 11.18112761 | 1.853183472 | 2.985306811 | 5.296541476 | | 1.666603649 | 0.043861753 | 0.999784719 |
| GLIDR | 37.77393184 | 44.12033862 | 41.67511199 | 24.09138514 | 20.89714767 | 28.60132397 | | 0.748067677 | 0.043870412 | 0.999784719 |
| PLD4 | 9.893172626 | 1.07610582 | 8.131729169 | 0.926591736 | 0.99510227 | 1.059308295 | | 2.692197137 | 0.043872122 | 1 |
| AL354919.2 | 9.893172626 | 4.30442328 | 7.115263023 | 0 | 2.985306811 | 1.059308295 | | 2.419587654 | 0.044038709 | 1 |
| DONSON | 342.6635246 | 407.8441057 | 331.3679636 | 312.2614151 | 274.6482266 | 277.5387734 | | 0.321956655 | 0.044080982 | 0.999784719 |
| Z98884.2 | 15.2894486 | 20.44601058 | 15.24699219 | 6.486142153 | 6.965715891 | 8.474466362 | | 1.218237257 | 0.044106231 | 0.999784719 |
| ATP7A | 247.3293156 | 214.1450582 | 247.0012735 | 320.6007407 | 284.5992493 | 282.8353148 | | -0.325487618 | 0.044285968 | 0.999784719 |
| OR7E38P | 2.698137989 | 8.608846559 | 0 | 0 | 0 | 0 | | 4.362626096 | 0.044511535 | 1 |
| ACAT2 | 606.1816682 | 647.8157036 | 548.8917189 | 492.0202119 | 546.3111463 | 425.8419347 | | 0.299516313 | 0.04455283 | 0.999784719 |
| GRHL1 | 34.17641453 | 40.89202116 | 36.59278126 | 25.94456861 | 22.88735221 | 13.77100784 | | 0.826396241 | 0.044637957 | 0.999784719 |
| AF131215.5 | 53.96275978 | 50.57697354 | 64.0373672 | 65.78801326 | 89.55920432 | 94.27843828 | | -0.563743291 | 0.044682405 | 0.999784719 |
| RBM4 | 293.1976615 | 256.1131851 | 230.7378152 | 189.9513059 | 232.8539312 | 179.0231019 | | 0.375121759 | 0.04469858 | 0.999784719 |
| ABCB1 | 195.1653145 | 195.8512592 | 209.3920261 | 263.152053 | 233.8490335 | 257.4119158 | | -0.329791665 | 0.044722537 | 0.999784719 |
| AL928654.4 | 70.15158771 | 60.26192592 | 70.13616408 | 44.47640333 | 43.78449989 | 46.60956499 | | 0.574411103 | 0.044843866 | 0.999784719 |
| UQCC2 | 407.4188363 | 399.2352592 | 344.5820235 | 348.3984928 | 292.5600674 | 277.5387734 | | 0.324905436 | 0.044937156 | 0.999784719 |
| LGALS9 | 1753.789693 | 2297.485926 | 1893.67643 | 2301.653872 | 2588.261005 | 2282.809376 | | -0.271282536 | 0.044964599 | 0.999784719 |
| LINC01238 | 9.893172626 | 4.30442328 | 4.065864584 | 1.853183472 | 0.99510227 | 0 | | 2.65206372 | 0.04497889 | 1 |
| ZXDA | 145.6994514 | 110.8388995 | 162.6345834 | 201.0704067 | 168.1722837 | 192.7941097 | | -0.421589291 | 0.045017663 | 0.999784719 |
| AL121917.1 | 0.89937933 | 1.07610582 | 5.082330731 | 12.04569257 | 6.965715891 | 8.474466362 | | -1.974641557 | 0.045117281 | 0.999784719 |
| SCAMP1-AS1 | 102.5292436 | 121.5999577 | 118.9265391 | 85.24643972 | 76.6228748 | 87.92258851 | | 0.456104664 | 0.045286805 | 0.999784719 |
| HOGA1 | 8.993793296 | 10.7610582 | 20.32932292 | 19.45842646 | 31.84327265 | 30.71994056 | | -1.034941848 | 0.045433384 | 0.999784719 |
| AC233699.1 | 24.2832419 | 45.19644444 | 19.31285678 | 16.67865125 | 6.965715891 | 18.00824102 | | 1.088336641 | 0.045480726 | 0.999784719 |
| RHAG | 4.496896648 | 1.07610582 | 3.049398438 | 11.11910083 | 8.955920432 | 9.533774658 | | -1.755957322 | 0.045530352 | 0.999784719 |
| RPL23AP2 | 1.798758659 | 7.532740739 | 3.049398438 | 0 | 0 | 1.059308295 | | 3.527335308 | 0.045564775 | 1 |
| VLDLR | 62.95655307 | 94.69731215 | 78.26789325 | 51.88913722 | 63.68654529 | 37.07579034 | | 0.622402857 | 0.04572133 | 0.999784719 |
| TNFAIP2 | 1000.109815 | 809.2315766 | 888.3914117 | 1201.789482 | 983.1610429 | 1083.672386 | | -0.276439017 | 0.045728864 | 0.999784719 |
| ADAMTSL4-AS1 | 2.698137989 | 4.30442328 | 10.16466146 | 0 | 0 | 2.118616591 | | 3.074483598 | 0.045734695 | 1 |
| PRND | 2.698137989 | 0 | 1.016466146 | 1.853183472 | 11.94122724 | 8.474466362 | | -2.528979275 | 0.045766713 | 1 |
| KNSTRN | 327.374076 | 397.0830475 | 354.746685 | 279.8307043 | 300.5208856 | 292.3690895 | | 0.305302718 | 0.045834312 | 0.999784719 |
| MIR100HG | 253.624971 | 319.6034285 | 272.4129272 | 214.9692828 | 230.8637267 | 223.5140503 | | 0.335872639 | 0.045838078 | 0.999784719 |
| CLSTN3 | 2267.33529 | 1712.084359 | 2261.637175 | 2403.578963 | 2446.956482 | 2736.193327 | | -0.281233953 | 0.046165784 | 0.999784719 |
| COL1A2 | 199.6622112 | 249.6565502 | 264.281198 | 251.1063605 | 339.3298741 | 341.0972711 | | -0.384970382 | 0.046238007 | 0.999784719 |
| LINC02018 | 0.89937933 | 3.22831746 | 5.082330731 | 0 | 0 | 0 | | 4.065914917 | 0.046332793 | 1 |
| AC020763.1 | 0.89937933 | 3.22831746 | 5.082330731 | 0 | 0 | 0 | | 4.065914917 | 0.046332793 | 1 |
| AL353708.1 | 0 | 0 | 0 | 0.926591736 | 3.980409081 | 4.237233181 | | -4.053059232 | 0.046457559 | 1 |
| LINC00348 | 0 | 0 | 0 | 0.926591736 | 3.980409081 | 4.237233181 | | -4.053059232 | 0.046457559 | 1 |
| AC069200.1 | 2.698137989 | 9.684952379 | 6.098796877 | 1.853183472 | 0.99510227 | 0 | | 2.651715527 | 0.046523999 | 1 |
| MICA | 914.6687782 | 1132.063323 | 859.9303596 | 765.364774 | 854.7928501 | 759.5240477 | | 0.288034923 | 0.046718844 | 0.999784719 |
| LINC02154 | 2.698137989 | 1.07610582 | 2.032932292 | 10.1925091 | 6.965715891 | 6.355849772 | | -2.002000449 | 0.046813978 | 1 |
| LINC01801 | 0 | 1.07610582 | 0 | 7.412733889 | 3.980409081 | 1.059308295 | | -3.550366967 | 0.047029016 | 1 |
| SLC15A3 | 150.196348 | 182.9379894 | 148.4040573 | 199.2172233 | 204.9910677 | 215.0395839 | | -0.364265315 | 0.047089307 | 0.999784719 |
| TCEA1P2 | 55.76151844 | 85.01235977 | 52.8562396 | 97.29213229 | 72.64246572 | 123.9390705 | | -0.604303039 | 0.047148202 | 0.999784719 |
| LINC01841 | 5.396275978 | 3.22831746 | 3.049398438 | 0 | 0.99510227 | 0 | | 3.457693649 | 0.047212326 | 1 |
| TNFRSF25 | 266.2162816 | 376.637037 | 307.9892423 | 276.1243374 | 222.9029085 | 235.1664416 | | 0.370328692 | 0.047521891 | 0.999784719 |
| LRRC32 | 6168.842822 | 4887.672634 | 6604.997017 | 7093.05974 | 6619.420301 | 7592.062552 | | -0.270434117 | 0.047687927 | 0.999784719 |
| CDRT4 | 5.396275978 | 13.98937566 | 25.41165365 | 5.559550417 | 0.99510227 | 7.415158067 | | 1.679987221 | 0.047885811 | 0.999784719 |
| CD59 | 14761.51294 | 19140.69422 | 15919.89278 | 12456.17271 | 14998.18142 | 14049.60592 | | 0.263507924 | 0.048099834 | 0.999784719 |
| CPT1A | 2139.623425 | 1810.009989 | 2411.057699 | 2441.569225 | 2475.814448 | 2729.837477 | | -0.265474168 | 0.048163011 | 0.999784719 |
| FBXO24 | 6.295655307 | 7.532740739 | 4.065864584 | 10.1925091 | 17.91184086 | 16.94893272 | | -1.330363021 | 0.04823822 | 0.999784719 |
| C1R | 74.64848436 | 83.93625395 | 78.26789325 | 101.925091 | 107.4710452 | 114.4052959 | | -0.451746764 | 0.048420322 | 0.999784719 |
| IFI27 | 739.2898089 | 887.7873014 | 687.1311148 | 984.9670155 | 967.2394066 | 856.9804109 | | -0.280540777 | 0.048470511 | 0.999784719 |
| AC098614.1 | 83.64227765 | 111.9150053 | 87.41608857 | 69.49438021 | 72.64246572 | 54.02472306 | | 0.52393931 | 0.04849382 | 0.999784719 |
| AC007952.1 | 1.798758659 | 6.456634919 | 1.016466146 | 0 | 0 | 0 | | 4.073922315 | 0.04852192 | 1 |
| PJVK | 36.87455251 | 32.2831746 | 51.83977345 | 31.50411903 | 15.92163632 | 20.12685761 | | 0.83575348 | 0.048532736 | 0.999784719 |
| RHD | 18.88696592 | 21.5221164 | 31.51045053 | 13.89887604 | 9.951022702 | 11.65239125 | | 1.013635313 | 0.048580873 | 0.999784719 |
| AC004925.1 | 3.597517318 | 4.30442328 | 1.016466146 | 0 | 0 | 0 | | 4.025713174 | 0.048700062 | 1 |
| CASKIN1 | 17.08820726 | 11.83716402 | 10.16466146 | 19.45842646 | 25.87265902 | 32.83855715 | | -0.988083565 | 0.04872968 | 0.999784719 |
| RARA-AS1 | 49.46586313 | 43.0442328 | 43.70804428 | 31.50411903 | 15.92163632 | 32.83855715 | | 0.764810033 | 0.048759638 | 0.999784719 |
| AL669942.1 | 8.993793296 | 0 | 2.032932292 | 0 | 0 | 0 | | 4.343235936 | 0.048764748 | 1 |
| VWF | 85423.04873 | 56885.10585 | 91183.1121 | 104652.977 | 85973.85084 | 105846.0849 | | -0.344524145 | 0.049017639 | 0.999784719 |
| KTN1-AS1 | 51.26462179 | 61.33803174 | 59.97150262 | 46.3295868 | 35.82368173 | 26.48270738 | | 0.660279979 | 0.04909766 | 0.999784719 |
| SPAG7 | 923.6625715 | 1212.771259 | 831.4693075 | 759.8052236 | 862.7536682 | 753.1681979 | | 0.320522009 | 0.049244917 | 0.999784719 |
| ANKMY1 | 150.196348 | 131.28491 | 148.4040573 | 113.9707835 | 101.5004316 | 110.1680627 | | 0.401540047 | 0.049815398 | 0.999784719 |
| PREX1 | 4783.798654 | 3578.051851 | 4694.040663 | 5551.211091 | 4808.334169 | 5434.251555 | | -0.274494304 | 0.049818261 | 0.999784719 |
| PCAT19 | 737.4910503 | 882.4067723 | 721.6909637 | 711.6224533 | 594.0760553 | 619.6953527 | | 0.281251667 | 0.049874848 | 0.999784719 |

| **Table S4. RNA-seq databases in MMD** | | | | | | |
| --- | --- | --- | --- | --- | --- | --- |
| Number | database | Tag | FPKM | Group | NormalizedFPKM | 2^(NormalizedFPKM) |
| 1 | GEO141022 | GSM4192549 | 0.681691329 | MMD | 0.869702431 | 1.827285967 |
| 2 | GEO141022 | GSM4192550 | 0.482726695 | MMD | -0.026515859 | 0.981788478 |
| 3 | GEO141022 | GSM4192551 | 0.400692582 | MMD | -0.396031139 | 0.759946026 |
| 4 | GEO141022 | GSM4192552 | 0.893585703 | MMD | 1.824161571 | 3.541011603 |
| 5 | GEO141022 | GSM4192553 | 0.265492714 | IA | -1.005026778 | 0.498260883 |
| 6 | GEO141022 | GSM4192554 | 0.895343133 | IA | 1.832077756 | 3.560494826 |
| 7 | GEO141022 | GSM4192555 | 0.551582622 | IA | 0.283639468 | 1.217261785 |
| 8 | GEO141022 | GSM4192556 | 0.107126503 | IA | -1.718373124 | 0.303891215 |
| 9 | GEO157628 | GSM4771802 | -0.169318018 | MMD | 0.083836513 | 1.059832676 |
| 10 | GEO157628 | GSM4771803 | -0.222256417 | MMD | 0.039752258 | 1.027937292 |
| 11 | GEO157628 | GSM4771804 | 2.851637852 | MMD | 2.599526286 | 6.060875831 |
| 12 | GEO157628 | GSM4771805 | 5.183225916 | MMD | 4.541147582 | 23.28207247 |
| 13 | GEO157628 | GSM4771806 | 4.192944088 | MMD | 3.71649407 | 13.14547228 |
| 14 | GEO157628 | GSM4771807 | 1.130317258 | MMD | 1.166102959 | 2.244047103 |
| 15 | GEO157628 | GSM4771808 | 6.93E-04 | MMD | 0.225412274 | 1.169111294 |
| 16 | GEO157628 | GSM4771809 | -0.067170864 | MMD | 0.168899176 | 1.124200355 |
| 17 | GEO157628 | GSM4771810 | -0.784034964 | MMD | -0.428066739 | 0.743257108 |
| 18 | GEO157628 | GSM4771811 | -1.052720808 | MMD | -0.651813877 | 0.636479575 |
| 19 | GEO157628 | GSM4771812 | 0.639718899 | MMD | 0.757558999 | 1.690627707 |
| 20 | GEO157628 | GSM4771813 | -1.416050765 | IA | -0.954375549 | 0.516064913 |
| 21 | GEO157628 | GSM4771814 | 0.625472824 | IA | 0.745695633 | 1.676782579 |
| 22 | GEO157628 | GSM4771815 | -0.48947658 | IA | -0.18277434 | 0.881007167 |
| 23 | GEO157628 | GSM4771816 | -1.914602787 | IA | -1.369542892 | 0.387013852 |
| 24 | GEO157628 | GSM4771817 | -4.083871431 | IA | -3.175993294 | 0.110644735 |
| 25 | GEO157628 | GSM4771818 | -3.536800447 | IA | -2.720421965 | 0.151729975 |

| **Table S5. Primers for rt-qPCR** | |
| --- | --- |
| Genes | Sequence |
| ITGB4-F | AAAGCGAGTACAGCAGCATCAC |
| ITGB4-R | GGTCAGCCCATCCACTAGGA |
| NOTCH1-F | GAGGCGTGGCAGACTATGC |
| NOTCH1-R | CTTGTACTCCGTCAGCGTGA |
| NOTCH2-F | CAACCGCAATGGAGGCTATG |
| NOTCH2-R | GCGAAGGCACAATCATCAATGTT |
| NOTCH3-F | TGGCGACCTCACTTACGACT |
| NOTCH3-R | CACTGGCAGTTATAGGTGTTGAC |
| NOTCH4-F | TGTGAACGTGATGTCAACGAG |
| NOTCH4-R | ACAGTCTGGGCCTATGAAACC |
| HES1-F | ATGACAGTGAAGCACCTCCG |
| HES1-R | CGTTCATGCACTCGCTGAAG |
| HES5-F | CATCAACAGCAGCATCGAGC |
| HES5-R | TGCTTCAGGTAGCTGACAGC |
| HEY1-F | GTTCGGCTCTAGGTTCCATGT |
| HEY1-R | CGTCGGCGCTTCTCAATTATTC |
| HEY2-F | AAGGCGTCGGGATCGGATAA |
| HEY2-R | AGAGCGTGTGCGTCAAAGTAG |
| ACTB-F | CCACCATGTACCCTGGCATT |
| ACTB-R | CGGACTCGTCATACTCCTGC |

| **Table S6. Sequence of siRNAs** | |
| --- | --- |
| Genes | Sequence |
| ITGB4 (human)-siRNA | CCACAGAGCUGGUGCCCUATT |
| Smad4 (huaman)-siRNA | CGGUCUUUGUACAGAGUUATT |

| **Table S7. The company and product number of all the antibodies** | | |
| --- | --- | --- |
| Antibody | Company | Product number |
| Vimentin | Cell Signaling Technology, USA | #5741 |
| αSMA | abcam, UK | ab124964 |
| VE-cadherin | Cell Signaling Technology, USA | #2500 |
| CD31 | Cell Signaling Technology, USA | #3528 |
| ITGB4 | abcam, UK | ab182120 |
| β-actin | Proteintech, USA | 20536-1 |
| phospho MEK | abcam, UK | ab278723 |
| MEK | abcam, UK | ab178876 |
| phospho P38 | Cell Signaling Technology, USA | #9216 |
| P38 | Cell Signaling Technology, USA | #8690 |
| phospho ERK | abcam, UK | ab201015 |
| ERK | abcam, UK | ab184699 |
| phospho JNK | abcam, UK | ab76572 |
| JNK | abcam, UK | ab208035 |
| phospho Smad1/5 | Cell Signaling Technology, USA | #9516 |
| Smad1/5 | abcam, UK | ab75273 |
| phospho Smad2/3 | Cell Signaling Technology, USA | #8828 |
| Smad2/3 | Cell Signaling Technology, USA | #8685 |
| β-catenin | abcam, UK | ab32572 |
| Ubiquitin | abcam, UK | ab134953 |
| Smad4 | abcam, UK | ab230815 |
| Ubiquitin K48 | abcam, UK | ab140601 |

**Supplementary Figures**

**
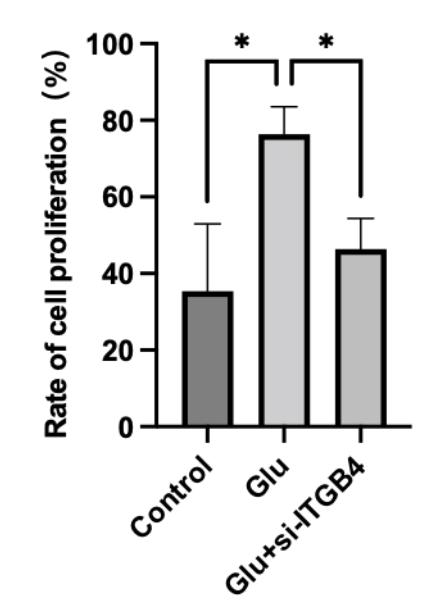
**

Figure S1. Histogram of the Edu assay in HBMECs subjected to glutamine treatment and ITGB4 knockdown. *P<0.05. HBMECs, human brain microvascular endothelial cells.


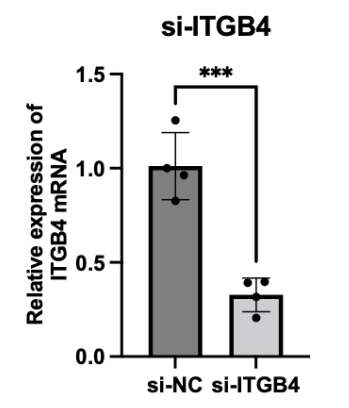


Figure S2. The knockdown efficiency of ITGB4 using ITGB4 siRNA in HBMECs. *P<0.05. HBMECs, human brain microvascular endothelial cells.


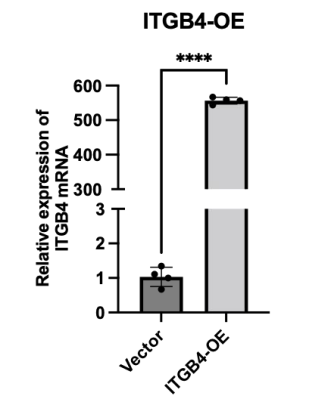


Figure S3. The overexpression efficiency induced by ITGB4 adenovirus in HBMECs. *P<0.05. HBMECs, human brain microvascular endothelial cells.


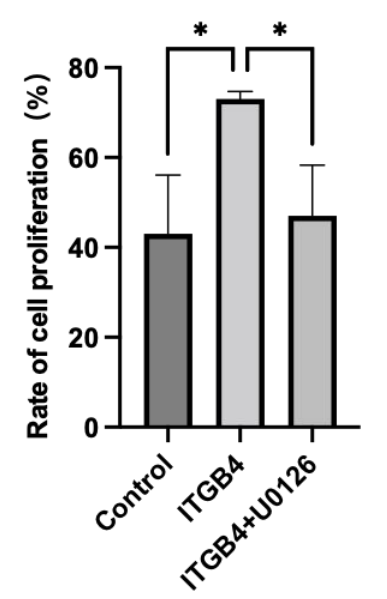


Figure S4. Histogram of the Edu assay in ITGB4-transfected HBMECs and U0126 treatment. *P<0.05. HBMECs, human brain microvascular endothelial cells.


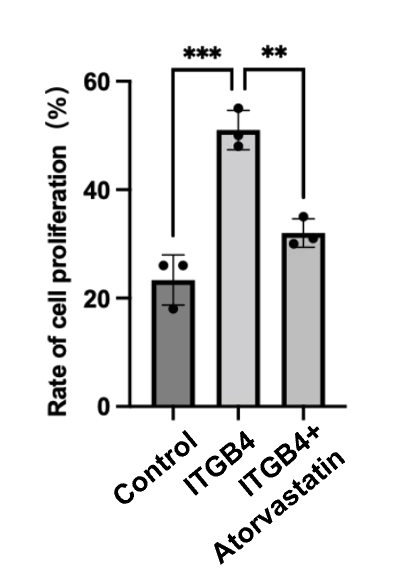


Figure S5. Histogram of the Edu assay in ITGB4-transfected HBMECs and Atorvastatin treatment. **P<0.01, ***P<0.001. HBMECs, human brain microvascular endothelial cells.


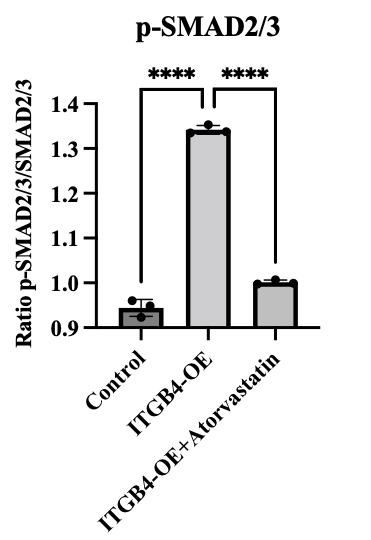


Figure S6. Histogram of the phosphorylation level of Smad2/3 in ITGB4-transfected HBMECs treated with Atorvastatin. ****P<0.0001. HBMECs, human brain microvascular endothelial cells.


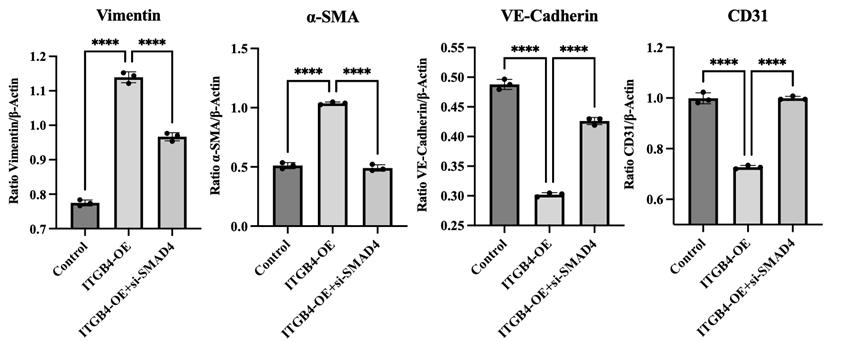


Figure S7. Histograms show the protein level of mesenchymal markers (Vimentin and αSMA) and endothelial markers (CD31 and VE-cadherin) in HBMECs after Smad4 knockdown. ***P<0.001, ****P<0.0001. HBMECs, human brain microvascular endothelial cells.
